# Ultrasoft and High‐Mobility Block Copolymers for Skin‐Compatible Electronics

**DOI:** 10.1002/adma.202005416

**Published:** 2020-12-14

**Authors:** Kristina Ditte, Jonathan Perez, Soosang Chae, Mike Hambsch, Mahmoud Al‐Hussein, Hartmut Komber, Peter Formanek, Stefan C. B. Mannsfeld, Andreas Fery, Anton Kiriy, Franziska Lissel

**Affiliations:** ^1^ Leibniz‐Institut für Polymerforschung Dresden e.V. Hohe Straße 6 Dresden 01069 Germany; ^2^ Faculty of Chemistry and Food Chemistry Technische Universität Dresden Dresden 01062 Germany; ^3^ Center for Advancing Electronics Dresden and Faculty of Electrical and Computer Engineering Technische Universität Dresden Helmholtzstraße 18 Dresden 01069 Germany; ^4^ Leibniz Institute for Solid State and Materials Research Helmholtzstraße 20 Dresden 01069 Germany; ^5^ Physics Department and Hamdi Mango Center for Scientific Research The University of Jordan Amman 11942 Jordan

**Keywords:** block copolymers, organic field‐effect transistors, skin‐compatible electronics, stretchable organic electronics

## Abstract

Polymer semiconductors (PSCs) are an essential component of organic field‐effect transistors (OFETs), but their potential for stretchable electronics is limited by their brittleness and failure susceptibility upon strain. Herein, a covalent connection of two state‐of‐the‐art polymers—semiconducting poly‐diketo‐pyrrolopyrrole‐thienothiophene (PDPP‐TT) and elastomeric poly(dimethylsiloxane) (PDMS)—in a single triblock copolymer (TBC) chain is reported, which enables high charge carrier mobility and low modulus in one system. Three TBCs containing up to 65 wt% PDMS were obtained, and the TBC with 65 wt% PDMS content exhibits mobilities up to 0.1 cm^2^ V^−1^ s^−1^, in the range of the fully conjugated reference polymer PDPP‐TT (0.7 cm^2^ V^−1^ s^−1^). The TBC is ultrasoft with a low elastic modulus (5 MPa) in the range of mammalian tissue. The TBC exhibits an excellent stretchability and extraordinary durability, fully maintaining the initial electric conductivity in a doped state after 1500 cycles to 50% strain.

## Graphical abstract

Triblock copolymers (TBCs) comprising a high‐performance polymer semiconductor endcapped with soft elastomeric chains can phase segregate at the nanoscale, while preserving the properties of the individual blocks. The TBCs are ultrasoft (5 MPa), electronically functional (0.1 cm^2^ V^−1^ s^−1^), stretchable and durable, and maintain conductivity after 1500 cycles to 50% strain in a doped state.

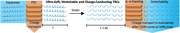

Polymer semiconductors (PSCs) are an integral part of organic thin‐film transistors (TFTs) for wearable devices (e.g., for health monitoring)^[^ ^1^, ^2^ ^]^ or implants,^[^ ^3^, ^4^ ^]^ for which they not only need to conduct charges but also have to be soft and flexible. Yet state‐of‐the‐art PSCs are brittle and prone to mechanical failure,^[^ ^5^, ^6^ ^]^ and, compared to a mammalian tissue, their elastic modulus (*E*_s_) is orders of magnitudes too high (cardiac muscle 8 kPa, skin 0.1–5 MPa, nerve 5 MPa;^[^ ^7^ ^]^ DPP‐based polymers 0.1–0.2 GPa;^[^ ^8^ ^]^ poly(3‐hexylthiophene)s (P3HT) 0.3–1.4 GPa^[^ ^9^ ^]^). Current research focuses on the synthesis of stretchable PSCs utilizing: (i) strain‐accommodating engineering,^[^ ^10^, ^11^, ^12^ ^]^ (ii) polymer composites^[^ ^13^ ^]^ and blends,^[^ ^14^, ^15^, ^16^, ^17^ ^]^ and (iii) molecular engineering.^[^ ^18^, ^19^ ^]^ Of these, only (ii) and (iii) might also yield PSCs with low *E*_s_.

Obtaining functional semiconducting polymer blends depends on choosing the best ratio and suitable processing method. Homogeneous blends of semiconducting and elastomeric components are insensitive to the gate voltage in conventional transistors. Instead, the PSC should ideally form an interpenetrating network at the interface formed with the dielectric layer.^[^ ^16^ ^]^ Blends of 1–3 wt% PH3T in polystyrene‐*block*‐poly(ethylene‐*co*‐butylene)‐*block*‐polystyrene (SEBS) have a low modulus in the range of pure SEBS (6.9–11.7 MPa), and the mobility of selected blends only decreased slightly from initially 6 × 10^−3^ to 2 × 10^−3^ cm^2^ V^−1^ s^−1^ under 50% strain after 200 cycles.^[^ ^16^ ^]^ To achieve this functionality, pretreatment of the P3HT solution to obtain nanofibril bundles is a prerequisite, and was accomplished by developing a specific cooling‐and‐heating protocol. Phase‐segregated, highly networked semiconducting architecture can also be achieved with blends of semiconducting polymers and poly(dimethylsiloxane) (PDMS): 0.49–0.75 wt% P3HT, pretreated with poor solvent/ultrasonification protocols, can be blended into a mixture of a PDMS elastomer resin and the suitable cross‐linking agent. Due to the formation of highly networked and dentritic‐like P3HT structures, the resulting films had improved mobilities (0.18 cm^2^ V^−1^ s^−1^, compared to 8.8 × 10^−2^ cm^2^ V^−1^ s^−1^ of the pristine polymer).^[^ ^20^ ^]^ Similarly, blends of 0.89 wt% PDPP‐TT also lead to improved mobilities (1.5 cm^2^ V^−1^ s^−1^, compared to 0.8 cm^2^ V^−1^ s^−1^ of the pristine polymer).^[^ ^21^ ^]^ The films maintain mobilities for up to 300 cycles to 100% strain, and the modulus can be assumed to be in the range of crosslinked PDMS.^[^ ^21^ ^]^

The blending approach thus yields highly functional hybrid materials, but also requires to carefully choose the blend ratios and develop processing procedures: for example, blends of 35–45 wt% PDPP‐TT in poly(*N*‐vinyl carbazole) are stable and show excellent mobilities, while loadings outside this range lead to undesired vertical or large lateral phase segregation.^[^ ^22^ ^]^ For blends of P3HT and elastomers, best results are achieved for much lower semiconductor contents (under 1 wt%), but require specific pretreatments of the P3HT to obtain phase‐segregated, highly networked semiconducting architectures.^[^ ^16^, ^20^, ^21^ ^]^

Molecular engineering on the other hands seeks to achieve the integration of different properties by introducing defined changes on the molecular level: Early routes focused on controlling molecular weight (MW) and dispersity (PDI), regioregularity and ordering,^[^ ^16^, ^18^ ^]^ main chain rigidity and side‐chain length.^[^ ^23^ ^]^ While these parameters influence *E*_s_, their overall impact is not sufficient to achieve a significant decrease. Recently, the focus shifted to introducing conjugation‐break spacers (CBS) into the backbone via random copolymerization. CBS decrease the backbone rigidity, and when the incorporated CBS are capable of forming weak and reversible intra‐ and interchain bonds, they can enable a PSC to be stretchable or to self‐heal.^[^ ^8^ ^]^ However, these materials still have high *E*_s_ (0.1–1 GPa)^[^ ^24^ ^]^ and the conjugation‐break spacer amount has to be kept low (<15%) as it is structurally tied to the reduction of the conjugation length (i.e., down to the dimer level for 50% CBS).

Block copolymers are well‐known materials used for various applications.^[^ ^25^ ^]^ When block copolymers consist of two or more thermodynamically incompatible polymers that are connected at their ends, a phase segregation into microphases with distinct properties can be achieved. Using this strategy for soft electronics, compromising between modulus and electrical performance can be avoided as the corresponding domains are spatially separated. Moreover, the material properties could be tuned by changing the size or type of the respective blocks. This approach was used to optimize the mechanical properties of electronically active materials for block copolymers based on P3HT: for instance, stretchability and charge mobility (0.02 cm^2^ V^−1^ s^−1^) was realized in a P3HT‐PE (polyethylene) diblock copolymer containing an impressive amount of 90% PE, nonetheless *E*_s_ could not be lowered below 0.24 GPa.^[^ ^26^ ^]^ A diblock copolymer of 30 wt% P3HT and poly(butyl acrylate) similarly showed a high modulus of 0.19 GPa, but was able to maintain mobilities of 2.5 × 10^−2^ cm^2^ V^−1^ s^−1^ under 100% strain for 1000 cycles.^[^ ^27^ ^]^ Combining 55 wt% P3HT with poly(methyl acrylate) in a triblock copolymer (TBC) on the other hand allowed to realize a lower modulus of 6 MPa, but the recorded initial mobility of 9 × 10^−4^ cm^2^ V^−1^ s^−1^ decreased significantly after 40% strain.^[^ ^28^ ^]^ Two sequences are possible for semiconducting triblock copolymers (TBCs), namely utilizing one inner PSC block or two outer PSC blocks. For TBCs of P3HT and poly(octylene oxide), it was found that while the former sequence had a slightly lower modulus (159 MPa compared to 195 MPa), it also had a much reduced stretchability, and a mobility that was one order of magnitude lower (7.5 × 10^−5^ cm^2^ V^−1^ s^−1^–2.5 × 10^−4^ cm^2^ V ^−1^s ^−1^).^[^ ^29^ ^]^ End‐capping P3HT with poly(δ‐decanolactone) gives a TBC with 8.5 × 10^−3^ cm^2^ V^−1^ s^−1^ but a high modulus of 1.46 GPa, and under 100% strain, only 9% of the mobility is retained.^[^ ^30^ ^]^

Here, we endcap a high‐performance donor–acceptor (D–A) PSC with mechanically compliant elastomeric units to obtain TBCs that are charge conducting, stretchable, and ultrasoft, that is, have moduli in the range of mammalian tissue (**Figure** **1**). Poly‐diketo‐pyrrolopyrrole‐thienothiophene (PDPP‐TT) was chosen as conjugated inner block, as this state‐of‐the‐art PSC has charge carrier mobilities exceeding P3HT by 2–3 orders of magnitude.^[^ ^21^, ^31^ ^]^ Linear PDMS chains combining outstanding mechanical properties (low *E*_s_, elastic deformation) with environmental stability and FDA approval,^[^ ^32^, ^33^ ^]^ were used as elastomeric endcaps. In the TBCs, lateral charge transport is possible through the PDPP‐TT blocks, while softness and flexibility are guided by the PDMS blocks. In a way, this design represents a covalently linked blend of a D–A PSC and an elastomer. From the molecular engineering viewpoint, the PDPP‐TT blocks can be understood as an extended conjugated system dynamically crosslinking the PDMS via π–π interactions.

**Figure 1:**
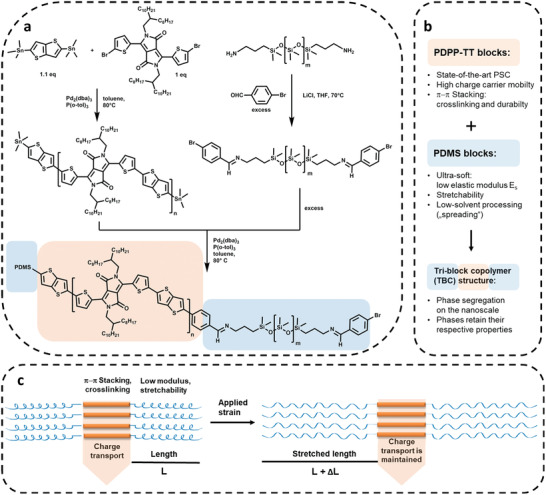
a) Synthesis of the polymeric building blocks and subsequent coupling. b) Summary of the properties of the respective blocks. c) Visualization of the phase segregation concept: charge transport is realized through the stacked inner PDPP‐TT blocks and maintained even when the elastic PDMS blocks are stretched under applied strain.

To obtain the TBCs, PDPP‐TT^[^ ^34^ ^]^ and modified PDMS were synthesized and subsequently coupled. All monomers used for the PDPP‐TT synthesis are either commercially available or were synthesized after literature protocols^[^ ^34^ ^]^ and characterized (Figures S1–S4, Supporting Information). For the reference polymer, the PSC block was endcapped with 4‐bromotoluene to fix the average MW and prevent further reactions (Figure S5, Supporting Information). When synthesizing PDPP‐TT blocks for the TBCs, an excess of thieno‐thiophene was used to control MW and obtain stannylated endgroups. For the elastomeric block, commercially available bis(3‐aminopropyl)‐terminated PDMS (number‐average molecular weight (*M*_n_) (PDMS) = 1, 2.5, and 25 kg mol^−1^) was modified with 4‐bromobenzaldehyde through an amine‐carbonyl condensation (Figure S6, Supporting Information). Finally, the PDPP‐TT block was coupled with an excess of modified PDMS under Stille conditions to obtain three TBCs (PDPP‐TT–PDMS‐1k/2.5k/25k).

The successful coupling was confirmed by high‐temperature ^1^H NMR spectroscopy. The reaction of the 4‐bromophenyl group with the stannylated end group of the PDPP‐TT block results in an additional set of signals representing the connecting group between both blocks. These signals are significantly broadened even at 120 °C (Figures S7–S9, Supporting Information) due to the restricted mobility of this moiety, resulting from a partial aggregation of neighboring PDPP‐TT blocks. The absence of stannyl end group signals and the presence of narrow signals of unreacted PDMS end groups prove the predominating triblock structure. Based on suitable signal integrals of the repeating units DPP‐TT (methyl group signal of the side chains) and PDMS (SiCH_3_ signal), the molar ratio of both co‐monomers in the TBC and also the wt% of PDPP‐TT and PDMS blocks in the final TBC were determined (24 wt%, 37 wt% and 65 wt% PDMS content for PDPP‐TT–PDMS‐1k/2.5k/25k, respectively). In high‐temperature gel permeation chromatography (HT‐GPC), a comparably high weight‐average molecular weight (M_w_) is detected for the reference PDPP‐TT (Table S1, Supporting Information), consistent with the ubiquitous M_w_ overestimation for conjugated polymers due to the deviation from the standard coil model^[^ ^35^ ^]^ and the aggregation also observed in the NMR spectra. Melting and crystallization temperatures were determined with differential scanning calorimetry (DSC) (Figures S10–S13, Supporting Information). Due to the high thermal stability of PDPP‐TT,^[^ ^36^ ^]^ no peaks associated with the conjugated block were detected, but all TBCs exhibited melting and crystallization temperatures typical for organosilicon rubbers,^[^ ^37^ ^]^ indicating phase separation within the TBC structure.^[^ ^38^ ^]^

Thermal stabilities were determined by thermal gravimetric analysis (TGA). All TBCs possess a two‐stage degradation curve with a first decomposition (defined at 5% weight loss) at 370 °C, and a second at 590 °C (Table S2 and Figure S14, Supporting Information). Consistent with the DSC results, TGA suggests the preservation of the building blocks’ properties within the TBC structure.

UV–Vis measurements of all polymers show the same absorption maxima in solution and in thin films (Table S3 and Figure  S15, Supporting Information). Likewise, all polymers exhibit a similar electrochemically irreversible behavior in cyclic voltammetry experiments. Both optical and electrochemical properties are defined by the inner PDPP‐TT block, while the outer PDMS blocks have no significant influence (Table S3, Figures S15, S16, Supporting Information).

Molecular packing, crystallinity, and chain orientation of all polymer films were investigated using grazing‐incidence wide‐angle X‐ray scattering (GIWAXS) of spin‐coated films. As can be seen in **Figures** **2**c and Figure S17, Supporting Information, PDPP‐TT exhibits a series of (*h*00) reflections up to the 4th order, typical of a lamellar morphology. The appearance of these lamellar reflections in the out‐of‐plane direction and the π–π stacking reflection (010) at *q* = 1.60 Å^−1^ in the in‐plane direction indicates a predominantly edge‐on orientation with a lamellar spacing d_100_ of 19.72 Å. A broad amorphous halo is also observed at *q* ≈ 1.33 Å^−1^, indicating the semicrystalline structure of the PDPP‐TT polymer. The GIWAXS patterns of both PDPP‐TT–PDMS‐1k and PDPP‐TT–PDMS‐2.5k films exhibit essentially the same features (Figures S18–S20, Supporting Information). In addition to the out‐of‐plane lamellar reflections and a weak in‐plane (010) reflection of the PDPP‐TT block, an amorphous halo corresponding to the PDMS blocks is observed at *q* ≈ 0.82 Å^−1^ in the copolymer films. The first‐order lamellar reflection exhibits less intensity with weak second order reflection implying less order of the PDPP‐TT domains. Contrarily, the PDPP‐TT–PDMS‐25k film exhibits a predominantly face‐on orientation of the PDPP‐TT crystalline lamellae (Figure S21, Supporting Information). The relative degree of crystallinity χ decreases with the PDMS content from 84.5% (PDPP‐TT, 0 wt% PDMS) to 71.5% (PDPP‐TT–PDMS‐1k, 24 wt% PDMS), 61.4% (PDPP‐TT–PDMS‐2.5k, 37 wt% PDMS), and finally 13.5% (PDPP‐TT–PDMS‐25k, 65 wt% PDMS). The lamellar spacing increases from 19.72 Å for the PDPP‐TT to 20.74 Å for PDPP‐TT–PDMS‐25k (Table S4, Supporting Information). When introducing more PDMS in the copolymer, larger PDMS domains are expected to form at the expense of the PDPP‐TT domains in the film. These amorphous domains impose limiting constrains on the PDPP‐TT chains and suppress their crystallization.

**Figure 2:**
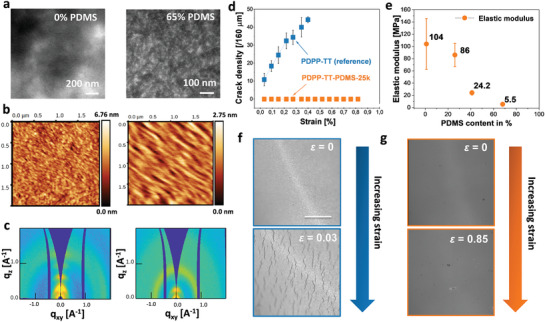
a) TEM images of (left) unstained reference PDPP‐TT and (right) PDPP‐TT–PDMS‐25k stained with RuO_4_. The contrast in the right image corresponds to the nanophase separation in the TBC, whereas the contrast in the left image corresponds to film thickness variation. b) AFM height images of (left) PDPP‐TT and (right) PDPP‐TT–PDMS‐25k sheared at 1 mm s^−1^. c) GIWAXS patterns of (left) PDPP‐TT and (right) PDPP‐TT–PDMS‐25k. The isotropic peak in the right image corresponds to the PDMS block. d) Variation in crack density upon increasing the applied strain for the reference PDPP‐TT and PDPP‐TT–PDMS‐25K TBC; e) Elastic modulus of the reference PDPP‐TT and TBCs (PDPP‐TT–PDMS‐1k/2.5k/25k) identified by nanoindentation method. The error bars correspond to the standard deviation due to the softness of the tip. f,g) Microscopy images of strained PDPP‐TT and PDPP‐TT–PDMS‐25k, respectively (scale bars denote 50 µm and ε is the amount of applied strain).

The surface morphology of solution‐sheared films was investigated by tapping mode atomic force microscopy (AFM). PDPP‐TT exhibits an interconnecting fiber‐like polymer chain network consistent with prior reports.^[^ ^39^, ^40^ ^]^ Both PDPP‐TT–PDMS‐1k and PDPP‐TT–PDMS‐2.5k possess a similar surface morphology due to the relatively low PDMS content (Figure S22, Supporting Information). This structure is no longer observable for PDPP‐TT‐PDMS‐25k, which instead shows a comparatively smooth surface (Figure 2b). All films were further investigated with transmission electron microscopy (TEM), and a nanophase separation was observed (Figures 2a; Figure S23, Supporting Information) with increasing domain sizes matching the increased PDMS content.

AFM nanoindentation showed a significant *E*_s_ decrease by three orders of magnitude, from 0.1 GPa for PDPP‐TT to 5 MPa for PDPP‐TT–PDMS‐25k (Figure 2e). This technique offers relative *E*_s_ values, allowing to compare the results with PDMS rubber (around 1 MPa)^[^ ^41^ ^]^ and common PSCs, for example, P3HT (0.1–1 GPa).^[^ ^24^ ^]^ Due to the relatively soft cantilever, the uncertainties are more pronounced for the hard reference PDPP‐TT and negligible for the soft TBCs.

To assess the film morphology under strain, the soft contact lamination method was applied:^[^ ^42^ ^]^ films of PDPP‐TT–PDMS‐25k and the reference PDPP‐TT polymer were analyzed with optical microscopy and AFM during uniaxial deformation using a home‐built stretching device (**Figure** **3**d). For PDPP‐TT, crack initiation was observed after 1% strain, and crack density increased linearly with applied strain at small strains (<21%), typical for a brittle material and consistent with earlier studies on PDPP‐TT.^[^ ^43^ ^]^ PDPP‐TT–PDMS‐25k on the other hand remained crack‐free up to a strain value of 85% (Figure 2f,g; Figure S24, Supporting Information). The enhanced stretchability was further demonstrated on a smaller length scale using AFM: here, PDPP‐TT–PDMS‐25k maintains a crack free surface up to 80% strain (Figure S25, Supporting Information).

**Figure 3:**
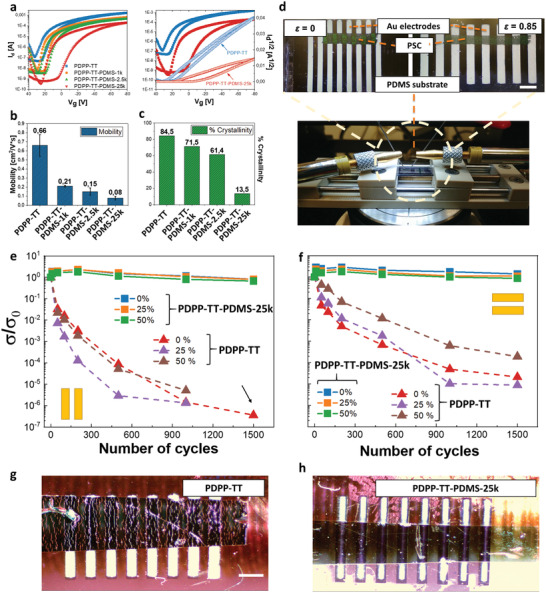
a) (left) Transfer characteristics of reference (PDPP‐TT) and TBCs (PDPP‐TT–PDMS‐1k/2.5k/25k) prepared by shearing at 1 mm s^−1^ (*V*_sd_ = ‐80 V), and (right) direct comparison of the transfer characteristics of PDPP‐TT and PDPP‐TT–PDMS‐25k. b,c) Comparison of reference (PDPP‐TT) and PDPP‐TT–PDMS‐25k regarding charge carrier mobilities (b) and crystallinity (%) (c). d) Photo of set up to measure conductivity under strain; scale bar: 1 mm. e,f) DC conductance of doped films of PDPP‐TT and PDPP‐TT–PDMS‐25k under various strains while stretching (ε = 0 to ε = 0.5), while increasing the number of stretching cycles up to 1500: e) parallel and f) perpendicular to the channel direction. The black arrow indicates the disconnection of two electrodes. g,h) Photos showing the film appearance of PDPP‐TT (g) and PDPP‐TT–PDMS‐25k (h) at 50% strain.

Bottom‐gate, top‐contact (BGTC) organic field‐effect transistors (OFETs) were fabricated via solution‐shearing to analyze the effect of the PDMS building block on the overall transistor performance.

The non‐ideality of the transfer curves (Figure 3a) complicates the mobilities calculations by using the traditional approach of fitting a slope to the curve. Specifically, gated contact behavior was previously identified to cause such “kinked” transfer curves, which, when analyzed in the traditional fashion, can lead to a serious overestimation of the mobility values.^[^ ^44^, ^45^ ^]^ Instead, we report effective field‐effect mobility values generated using the algorithm proposed by Choi et al.^[^ ^46^ ^]^ which accounts for non‐ideal curves and any non‐zero threshold voltage. This allows for a better comparison of the different materials reported herein as well as with literature values of similar materials. It was previously shown that OFETs made from very similar DPP‐based polymers grown on octadecyltrimethoxysilane (ODTMS) suffer from high positive threshold voltages.^[^ ^47^ ^]^ However, despite the growth on ODTMS, in our case the transfer curves are shifted toward more negative gate voltage values upon increasing the PDMS content. This might indicate an increasing degree of screening of the dipoles introduced by the ODTMS layer as the amount of PDMS in the polymer goes up.^[^ ^47^ ^]^

PDPP‐TT–PDMS‐1k (0.21 cm^2^ V^−1^ s^−1^) and PDPP‐TT–PDMS‐2.5k (0.15 cm^2^ V^−1^ s^−1^) showed saturated average charge carrier mobility (μ_sat,average_) values of the same order of magnitude as neat PDPP‐TT (0.66 cm^2^ V^−1^ s^−1^). Despite containing 65 wt% PDMS, PDPP‐TT–PDMS‐25k showed a high μ_sat,average_ of 0.08 cm^2^ V^−1^ s^−1^, confirming that semicrystalline domains of the semiconducting inner part are still interconnected (Figure 3a–b; Table S5, Supporting Information). The covalent connection between elastomer and PSC is an obligate prerequisite: physical blends of PDPP‐TT and PDMS were prepared containing the same ratios of both units as in the synthesized TBCs. No transfer curve was observed for these devices (Figure S26, Supporting Information), potentially due to an enrichment of PDMS at the top surface with sufficient mixed phase below the percolation threshold in the channel.

To investigate the electrical property under strain, we performed strain‐dependent DC conductivity measurements of doped PDPP‐TT–PDMS‐25k and PDPP‐TT films. Tetrafluoro‐7,7,8,8‐tetracyanoquinodimethane (F4‐TCNQ) was added to the polymer solutions to ensure a measurable DC conductivity.

Upon applying a uniaxial strain perpendicular to the channel direction, the conductivity of PDPP‐TT decreased in both stretching directions (Figure S28, Supporting Information). A strong crack formation is observed (Figure 3g; Figure S27, Supporting Information), which likely helps to maintain conduction path in perpendicular direction: here, the conductivity of PDPP‐TT film gradually decreased by one order of magnitude when increasing the strain up to 100%. For 100% strain in parallel direction, a decrease by two orders of magnitude is observed. In contrast, PDPP‐TT–PDMS‐25k exhibited constant conductivity values up to 100% strain in both perpendicular and parallel directions (Figure S28, Supporting Information), consistent with the results of the mechanical stretchability tests and the smooth and crack‐free appearance of the film (Figure 3h).

We then cycled both polymers up to 1500 times, loading and unloading with 0–50% strain. The conductivity was measured after selected cycle numbers (50, 100, 500, 1000 and 1500) at 0, 25 and 50% strain range. The addition of F4‐TCNQ can induce ductility in DPP‐based polymers,^[^ ^48^ ^]^ which might contribute to maintaining electrical functionality while stretching. Still, the conductivity of PDPP‐TT decreased steeply when the film was stretched repeatedly in either parallel (Figure 3e) or perpendicular (Figure 3f) direction. On the other hand, the TBC was able to fully maintain its initial electrical conductivity for 1500 cycles (Figure 3e,f). Interestingly, PDPP‐TT–PDMS‐25k shows isotropic behavior in both perpendicular and parallel directions; no decrease in conductivity is recorded for 1500 cycles to 50% strain.

In rotational rheology experiments, PDPP‐TT behaves like a Newtonian fluid in the investigated range of shear rates, while the TBCs show shear‐thinning behavior (see Figure S30, Supporting Information). Unlike typical PSCs, the TBCs do not dissolve instantly in organic solvents but rather swell, becoming soft and ductile, and enabling a low‐solvent and near waste‐free deposition method by spreading the soaked materials on hard surfaces (Figure S31, Supporting Information). PDPP‐TT–PDMS‐1k and PDPP‐TT–PDMS‐2.5k were spread on prefabricated BGBC OFET devices. While yielding relatively thick and non‐homogeneous films, the recorded mobilities are comparable to BGBC devices obtained via spin‐coating (Figure S32, Supporting Information).

In summary, we synthesized TBCs covalently endcapping a high‐performance D–A PSC with two elastomeric and insulating organosilicon blocks to combine favorable electrical and mechanical properties in one system. The chosen synthetic pathway leads to the nanophase segregation of both components, while preserving the features of both moieties (PDPP‐TT and PDMS) in their respective domains. The TBCs are ultrasoft and durable: the TBC with the highest PDMS content (65 wt%) has a low modulus (5 MPa) in the range of mammalian skin (0.1–5 MPa) and shows no crack formation up to 85% strain.

The TBCs possess swelling abilities, enabling a novel deposition approach by spreading the materials on a chip using only trace amounts of solvents. The TBC with 65 wt% PDMS achieves high charge carrier mobilities (≈0.1 cm^2^ V^−1^ s^−1^) in the range of the fully conjugated reference polymer PDPP‐TT (≈0.7 cm^2^ V^−1^ s^−1^). In a doped state, the TBC preserves full electronic functionality under prolonged mechanical stress: after stretching to 50% strain for 1500 cycles, no decrease in conductivity is observed compared to the initially recorded values in both parallel and perpendicular direction. Overall, we have detailed a strategy to achieve highly durable, electronically functional, stretchable and ultrasoft polymers by covalently connecting blocks with different physical properties.

## Conflict of Interest

The authors declare no conflict of interest.

## Supporting information

Supporting Information
