# Supplementary material for: Ultrasoft and High‐Mobility Block Copolymers for Skin‐Compatible Electronics
Source: Adv Mater. 2020 Dec 14;33(4):2005416. doi: 10.1002/adma.202005416 (PMC11468703; doi:10.1002/adma.202005416)
Supplement: Supplementary file 1 — Supporting Information [file ADMA-33-2005416-s001.pdf]

© 2020 Wiley-VCH GmbH

ADVANCED MATERIALS

Supporting Information

for *Adv. Mater.*, DOI: 10.1002/adma.202005416

Ultrasoft and High-Mobility Block Copolymers for Skin-Compatible Electronics

*Kristina Ditte, Jonathan Perez, Soosang Chae, Mike Hambsch, Mahmoud Al-Hussein, Hartmut Komber, Peter Formanek, Stefan C. B. Mannsfeld, Andreas Fery, Anton Kiriy, and Franziska Lissel**

Supporting Information

Ultrasoft and High-Mobility Block Copolymers for Skin-Compatible Electronics

Kristina Ditte[1,2], *Jonathan Perez*[2,3,4], *Dr. Soosang Chae*[1], *Dr. Mike Hambsch*[2,3], *Prof. Dr. Mahmoud Al-Hussein*[1,5], *Dr. Hartmut Komber*[1], *Dr. Peter Formanek*[1], *Prof. Dr. Stefan C. B. Mannsfeld*[2,3], *Prof. Dr. Andreas Fery*[1,2], *Dr. Anton Kiriy*[1] *and Dr. Franziska Lissel*[1,2*]

[1]Leibniz-Institut für Polymerforschung Dresden e.V., Hohe Straße 6, Dresden 01069, Germany [2]Faculty of Chemistry and Food Chemistry, Technische Universität Dresden, Dresden 01062, Germany

[3]Center for Advancing Electronics Dresden and Faculty of Electrical and Computer Engineering, Technische Universität Dresden, Helmholtzstraße 18, Dresden 01069, Germany [4]Leibniz Institute for Solid State and Materials Research, Helmholtzstraße 20, 01069 Dresden, Germany

[5]Physics Department and Hamdi Mango Center for Scientific Research, The University of Jordan, Amman 11942, Jordan

*E-mail: lissel@ipfdd.de

Contents

Ultra-soft and high mobility block-copolymers for skin-compatible electronics S1

1. Experimental Methods and Materials S4

1.1 Instrumentation.. S4

Nuclear Magnetic Resonance (NMR) Spectroscopy.. S4

High Temperature Gel Permeation Chromatography (HT-GPC) S4

Ultraviolet-visible (UV/Vis) Spectroscopy.. S4

Cyclic voltammetry (CV)... S5

Differential scanning calorimetry (DSC) S5

Thermogravimetric analysis (TGA) S5

Grazing Incidence Wide Angle X-ray Scattering (GIWAXS).. S6

Atomic Force Microscopy (AFM) S6

AFM: Nanoindentation S6

Optical Microscopy: Crack-onset S7

Transmission Electron Microscopy (TEM).. S7

Fabrication and characterization of OFET devices S8

Conductivity under strain measurements S8

Optical hot stage microscopy S9

Rheology S9

1.2 Materials.. S10

Chemicals S10

Precursor Syntheses... S10

Polymer Syntheses S13

2. Characterization Data S15

Nuclear Magnetic Resonance (NMR) Spectroscopy... S15

Differential Scanning Calorimetry (DSC)... S22

Thermogravimetric Analysis (TGA) S26

Ultraviolet-visible (UV/Vis) Spectroscopy S28
Cyclic Voltammetry (CV) S29
Grazing Incidence Wide Angle X-ray Scattering (GIWAXS) S31
Atomic Force Microscopy (AFM) S34
Transmission Electron Microscopy (TEM) S35
Microscopy Under Strain S36
3. Electrical Characterization S37
BGTC and BGBC Transfer Characteristics S37
4. Conductivity under strain S39
5. Low Solvent Processability (“Spreading”) S42
Rheology S42
Spreading Experiments S43
Electrical Characterization: BGBC transfer curves of spread and spin-coated films S44
6. References S45

1. Experimental Methods and Materials

1.1 Instrumentation.

Nuclear Magnetic Resonance (NMR) Spectroscopy

A Bruker Avance III 500 spectrometer was used to record ^{1}H NMR spectra at 500.13 MHz. The spectra were referenced to the residual solvent signals ($CDCl_3$: $\delta(^1H)$ = 7.26 ppm, DMSO-*d*$_6$: $\delta(^1H)$ = 2.50 ppm) and measured at 30°C. High-temperature NMR spectra were recorded at 120 °C and referenced to tetrachloroethane-d_2 ($C_2D_2Cl_4$: $\delta(^1H)$ = 5.98 ppm). Abbreviations were used for ^{1}H NMR spectra data as listed: s - singlet, d - doublet, dd - doublet of doublet, t - triplet, m -multiplet and br - broad signal.

High Temperature Gel Permeation Chromatography (HT-GPC)

HT-GPC measurements were performed against polystyrene (PS) standards using an Agilent PL-GPC 220 with two MIXED-B-LS columns. 1,2,4-Trichlorobenzene was used as eluent at 150 °C and the GPC set to a 1 mL/min flow rate. Polymer samples were dissolved (2.5 - 3.0 mg/mL) at 100 °C for at least 10 h prior to the measurements. UV-Detector: UVD-2L (UV-1 386 nm und UV-2 500 nm) from TESTA Analytical Solutions.

Ultraviolet-visible (UV/Vis) Spectroscopy

UV/Vis measurements were carried out on a Perkin Elmer “Lamda800” instrument at room temperature. Diluted solutions (c ~ 1 mg/mL) were prepared from chlorobenzene. For thin film UV/Vis measurements, polymer samples were spin-coated from chlorobenzene (c ~ 5 mg/mL) on glass slides.

Cyclic voltammetry (CV)

CV measurements were performed under inert atmosphere using a deoxygenated 0.1 M solution of tetra-*n*-butylammonium hexafluorophosphate (NBu_4PF_6) in acetonitrile as electrolyte. The three-electrode system was comprised of a non-aqueous silver/silver ion (Ag/Ag^+) reference electrode (silver wire in acetonitrile containing 0.1 mol/L NBu_4PF_6 and 0.01 mol/L $AgNO_3$), a platinum wire as counter electrode and a platinum disk as working electrode. A freshly prepared polymer film was deposited on the working electrode prior to each measurement by drop casting (c ~ 0.1 mg/mL). The voltagramms were recorded and referenced to the ferrocene/ferrocenium (Fc) redox couple as an external standard. HOMO and LUMO energies were calculated according to the following equations:

$$E_{HOMO} = -(5.09 + E_{onset,ox} - E_{\frac{1}{2},Fc})[eV] \quad (1)$$

$$E_{LUMO} = -(5.09 + E_{onset,red} - E_{\frac{1}{2},Fc})[eV] \quad (2)$$

Differential scanning calorimetry (DSC)

DSC measurements were performed on a TA Instruments Q1000 at a scan rate of 10 K/min under nitrogen atmosphere.

Thermogravimetric analysis (TGA)

TGA was performed on a TA Instruments Q5000 applying a nitrogen gas flow and a heating rate of 10 K/min.

Grazing Incidence Wide Angle X-ray Scattering (GIWAXS)

GIWAXS measurements were performed using a GANESHA 300XL+ system (SAXSLAB ApS, Lyngby/Denmark). The instrument was equipped with a Pilatus 300K detector, a Cu X-ray source operated at 50 kV/0.6 mA (λ = 1.5408 Å), and a three-slit collimation system. The detector was moved in a vacuum chamber to a sample-to-detector distance of 110 mm. The data were analyzed using GIXSGUI program. An incident angle of 0.2 ° was used. The relative degree of crystallinity χ and the lamellar spacing were calculated from radially integrated line profiles of the GIWAXS patterns. The whole curve was fitted using individual Gaussian functions for each peak and the amorphous broad amorphous hump extending between q =1.1 to 2.0 Å^{-1} superimposed on a linear background. All χ values were obtained from the ratio of the area under the (100) peak to that of the sum of the areas under the amorphous hump, extending between q =1.1 to 2.0 Å^{-1}, and the (100) peak. The lamellar spacing was calculated from the (100) peak position, q_{100}, according to following equation: $d_{100} = \frac{2\pi}{q_{100}}$.

Atomic Force Microscopy (AFM)

The surface morphology of the copolymer films was probed by atomic force microscopy using a Nanosurf Flex-Axiom equipped with Tap 190AI-G silicone tip in tapping mode. Nanosurf 300 was used as an analysis software.

AFM: Nanoindentation

Nanoindentation measurements were performed on a Dimension FastScan (Bruker-Nano, USA) with a SAA-HPI-30 (Bruker-Nano, USA) cantilever/tip, with a tip radius 30 nm and a spring constant of 0.286 N/m. At least 9 force-indention curves were taken from each sample in a 3×3 array with d_x, d_y = 300 nm. The elastic modulus was calculated after baseline correction

using the software NanoScope Analysis 2.0 (Bruker-Nano, USA) and the JKR model and taking adhesion forces into account, at an indentation depth of 3....4 nm. The calibration was checked by measuring a PDMS sample with E = 2.5 MPa.

Optical Microscopy: Crack-onset

For the investigation of crack initiation strain, the copolymer films were transferred on a PDMS substrate, which was cured at 80 °C for 1 hour with the 10 :1 mixing ratio of curing agent to base polymer (Sylgard 184, Dow corning), and optical microscopy (MarSurf CM expert, Nanofocus) was performed to obtain the crack images of the polymer film. To apply uniform uniaxial strain on the sample, a custom-made 1-D motorized stretching equipment was mounted under the microscope to conduct the measurement. The crack density of the polymer films was evaluated by counting the number of cracks along the cross-section profile in three different lines.

Transmission Electron Microscopy (TEM)

Thin films of TBC were spin coated on glass coated with thin PEDOT:PSS sacrificial layer. The PEDOT:PSS was dissolved in deionized water and pieces of TBC film floating on water surface were collected on TEM grids and stained for 5 min in RuO4 gas. The RuO4 stained the PDMS block, giving sufficient contrast in TEM. The images were recorded with Libra120 TEM (Carl Zeiss Microscopy GmbH, Germany) operated at 120 kV.

Fabrication and characterization of OFET devices

OFETs were fabricated on n-type doped Si (100) wafers with a 300 nm SiO2 thermally grown layer as a gate dielectric. The substrates were treated with octadecyltrimethoxysilane (ODTMS) according to the reported method[1]. The polymer solutions (25 mg/mL for PDMS-encapped polymers and 20 mg/mL for the PDPP-TT reference polymer) were prepared by dissolving the corresponding polymers in chlorobenzene and stirring at 80°C overnight. The polymer solutions were then either spin-coated at 1000 rpm for 60 sec or solution sheared. For solution shearing the substrate temperature (100 °C) was controlled by a thermocouple, and the coating speed was varied using a linear motor from Jenny Science. The blade angle was 8° with a gap of 100 um between the substrate and the edge of the blade. Bottom-gate, top-contact transistors were then finished by thermal evaporation of 50 nm-thick gold electrodes at a vacuum pressure of 10^{-7} mbar. Devices were electrically characterized under ambient conditions using a Keysight B1500 semiconductor analyzer, and mobilities were calculated according to Choi et al. [2]

Conductivity under strain measurements

Patterned gold electrodes on PDMS substrates were used as a stretchable substrate for the strain-dependent conductivity measurements. The PDMS substrates were cured at 80 °C for 1 hour with a 10:1 mixing ratio of curing agent to base polymer (Sylgard 184, Dow corning). Both polymer semiconductor films (PDPP-TT and PDPP-TT-PDMS-25000) were transferred from the OTMS treated Si wafer (spin-coated at 1000 rpm for 60 s) onto the stretchable electrode substrate. In order to increase the conductivity of the pristine polymer semiconductor film, 10% solution of F4-TCNQ in acetonitrile was added by different means prior to the DC conductivity measurements. Bulk doping: the dopant was added to the polymer solutions in 1(polymer):0.5(dopant) molar ratio. Interfacial doping: the dopant was spin-coated at 1000 rpm for 60 s. To apply precise uniaxial strain, the PDMS substrate with the polymer semiconductor film was mounted on the home-made motorized linear stage (L505, Physik Instrumente, PI

GmbH & Co. KG). A source measure unit (SMU) Keithley 2612B and a probe station (MPI TS50, MPI CORPORATION) were used to measure the current-voltage characteristics of the sample.

Optical hot stage microscopy

Optical polarized hot stage microscope observations were carried out on a ZEISS Axio Imager M2m. A slow heating to 350 °C was performed to determine the softening point of the polymers. Additionally, the processability of the polymers *via* spreading was tested by adding a drop of chlorobenzene.

Rheology

Rheological measurements were performed on a TA Instrument ARES-G2 rheometer with a cone plate attachment (diameter 25 mm, cone angle 0.1 rad, gap size 50 µm).

1.2 Materials

Chemicals

All used reagents and solvents were purchased from commercial suppliers and used without further purification, unless noted otherwise. *Bis*(3-aminopropyl) terminated poly(dimethylsiloxanes) were purchased from Sigma Aldrich and used without any further purification. All other monomers were synthesized according to literature protocols[3,4]. Polymerization was carried out under standard Stille cross-coupling conditions

Precursor Syntheses

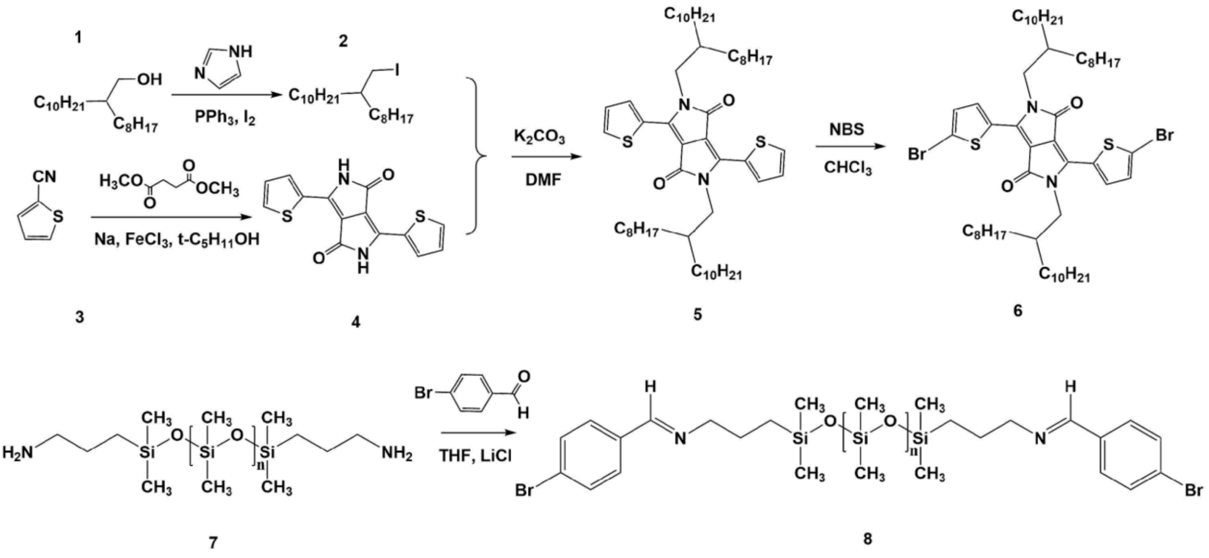

Scheme S1. Synthesis of precursors.

9-(Iodmethyl)nonadecane (2)

23.9 mL (67.1 mmol, 1.0 eq.) 2-octyl-1-dodecanol, 21.1 g (80.5 mmol, 1.2 eq.) triphenylphosphine and 5.5 g (80.5 mmol, 1.2 eq.) imidazole were dissolved in 115 mL dichloromethane (DCM). At 0 °C, 19.5 g (77.1 mmol, 1.15 eq.) iodine was added in portions to the reaction mixture. After stirring for 45 min at 0 °C, the ice bath was removed and the stirring maintained for 17 h before pouring the reaction mixture into an aqueous saturated

sodium thiosulfate solution. The aqueous layer was extracted twice with DCM and once with hexanes, the combined organic phases were washed with water and brine and dried over $MgSO_4$. Afterwards, the mixture was concentrated under reduced pressure and suspended in hexanes. The mixture was then filtered over a silica gel column and the product was obtained as a colorless oil.

Yield: 24.3 g (59.6 mmol, 90%)

^{1}H NMR (500 MHz, $CDCl_3$) δ [ppm] = 3.26 (d, J = 4.5 Hz, 2H), 1.35-1.15 (32H), 1.05 (m, 1H), 0.89 (t, J = 6.8 Hz, 6H).

3,6-Di(thiophen-2-yl)pyrrolo[3,4-*c*]pyrrole-1,4(2*H*,5*H*)-dione (4)

A three-neck flask was charged with 60 mL (0.55 mol, 13.7 eq.) anhydrous 2-methyl-2-butanol, 3.45 g (0.15 mol, 3.8 eq.) sodium and 40 mg (0.25 mmol, 0.01 eq.) anhydrous iron (III) chloride under nitrogen atmosphere, and the reaction mixture was stirred for 1 h at 110 °C until the sodium was completely dissolved. 10.9 g (0.10 mol, 2.5 eq.) 2-thiophenecarbonitrile was added at once followed by a dropwise addition of 5.8 g (0.04 mol, 1.0 eq.) dimethyl succinate in 5 mL 2-methyl-2-butanol over 30 min. After 2 h at 110 °C, the reaction mixture was cooled to 50 °C, diluted with 50 mL methanol and acidified with 15 mL glacial acetic acid. After refluxing for 5 min, the cooled reaction mixture was poured in 500 mL ice water, the precipitates were collected and washed with water and warm methanol. The final product was dried under vacuum at 40 °C yielding a brick-red solid.

Yield: 7.1 g (0.02 mol, 60%)

^{1}H NMR (500 MHz, DMSO-d_6) δ [ppm] = 11.21 (2H), 8.21 (2H), 7.95 (2H), 7.30 (2H).

2,5-*Bis*(2-octyldodecyl)-3,6-di(thiophen-2-yl)pyrrolo[3,4-*c*]pyrrole-1,4(2*H*,5*H*]-dione (5)

In 150 mL anhydrous dimethylformamide, 7.6 g (25.3 mmol, 1.0 eq.) 3,6-di(thiophen-2-yl)pyrrolo[3,4-c]pyrrole-1,4(2*H*,5*H*)-dione (**4**) and 0.35 g (1.27 mmol, 0.05 eq.) 18-crown-6 were dissolved at 120 °C before 12.3 g (88.7 mmol, 3.5 eq) anhydrous K_2CO_3 were added. After 1 h, 27.9 g (68.3 mmol, 2.7 eq.) 9-(iodmethyl)nonadecane (**2**) was added dropwise over 30 min and the reaction stirred for 20 h. The cooled reaction mixture was concentrated *in vacuo*, dissolved in ethyl acetate, washed with water and dried using $MgSO_4$. After removing the solvent under reduced pressure, the residue was dissolved in a small amount of chloroform and

precipitated into methanol. The collected precipitate was further purified *via* column chromatography using 2% ethyl acetate in hexanes resulting in a dark red solid.

Yield: 9.6 g (11.2 mmol, 44%)

^{1}H NMR (500 MHz, $CDCl_3$) δ [ppm] = 8.86 (d, *J* = 3.8 Hz, 2H), 7.61 (d, *J* = 5.0 Hz, 2H), 7.26 (dd, 2H), 4.02 (d, *J* = 7.7 Hz, 4H), 1.90 (m, *J* = 6.5 Hz, 2H), 1.45 – 1.10 (64H), 0.87 (2 x t, *J* = 7.2 Hz, 12H).

3,6-*Bis*(5-bromothiophen-2-yl)-2,5-*bis*(2-octyldodecyl)pyrrolo-[3,4-c]pyrrole-1,4(2*H*,5*H*)-dione (6)

To a solution of 5.6 g (6.5 mmol, 1.0 eq.) 2,5-*bis*(2-octyldodecyl)-3,6-di(thiophen-2-yl)pyrrolo[3,4-*c*]pyrrole-1,4(2*H*,5*H*]-dione (**5**) in 500 mL chloroform at 0 °C, 2.7 g (15.5 mmol, 2.39 eq.) *N*-bromosuccinimide was added in portions. After stirring for 16 h protected from light, the reaction solution was washed with water and dried over $MgSO_4$. The targeted material was isolated as a dark purple solid by column chromatography using 30% DCM in hexanes.

Yield: 5.4 g (5.3 mmol, 82%)

^{1}H NMR (500 MHz, $CDCl_3$) δ [ppm] = 8.62 (d, *J* = 4.2 Hz, 2H), 7.22 (d, *J* = 4.2 Hz, 2H), 3.92 (d, *J* = 7.7 Hz, 4H), 1.88 (m, 2H), 1.35 – 1.10 (64H), 0.87 (2 x t, *J* = 6.9 Hz, 12H).

(1*E*,1'*E*)-*N*,*N*'-((1,1,3,3-tetramethylpolysiloxane-1,3-diyl)*bis*(propane-3,1-diyl))*bis*(1-(4-bromophenyl)methanimine) (8)

Bis(3-aminopropyl) terminated polydimethylsiloxane (M_n= 2500 g/mol) (10 g, 4 mmol, 1 eq.), 4-bromobenzaldehyde (2.3 g, 12 mmol, 3 eq.) and lithium chloride (1.7 g, 40 mmol, 10 eq.) were dissolved in 25 mL of THF and heated to reflux. After 20 h, the reaction mixture was diluted with acetone, filtered through diatomite and the solvent was evaporated. The reaction mixture was re-dissolved in acetone, and 30 mL of methanol was added, followed by mixing, centrifugation and decantation of methanol from the reaction mixture. This procedure (mixing-centrifugation-decantation) was repeated 4-6 times, and the product was finally obtained as viscous yellow liquid after evaporating the solvent.

Yield: 9.5 g (3.6 mmol, 90%)

^{1}H NMR (500 MHz, $CDCl_3$) δ [ppm] = 8.21 (s, 2H), 7.60 (d, *J* = 8.5 Hz, 4H), 7.54 (d, *J* = 8.4 Hz, 4H), 3.60 (td, *J* = 7.1, 1.3 Hz, 4H), 1.74 (m, 4H), 0.58 (m, 4H), 0.27 – 0.10 (s, 350H).

Modified PDMS with other molecular weights (M_n= 1000 and 25000 g/mol) were synthesized following the procedure described for **8**.

Polymer Syntheses

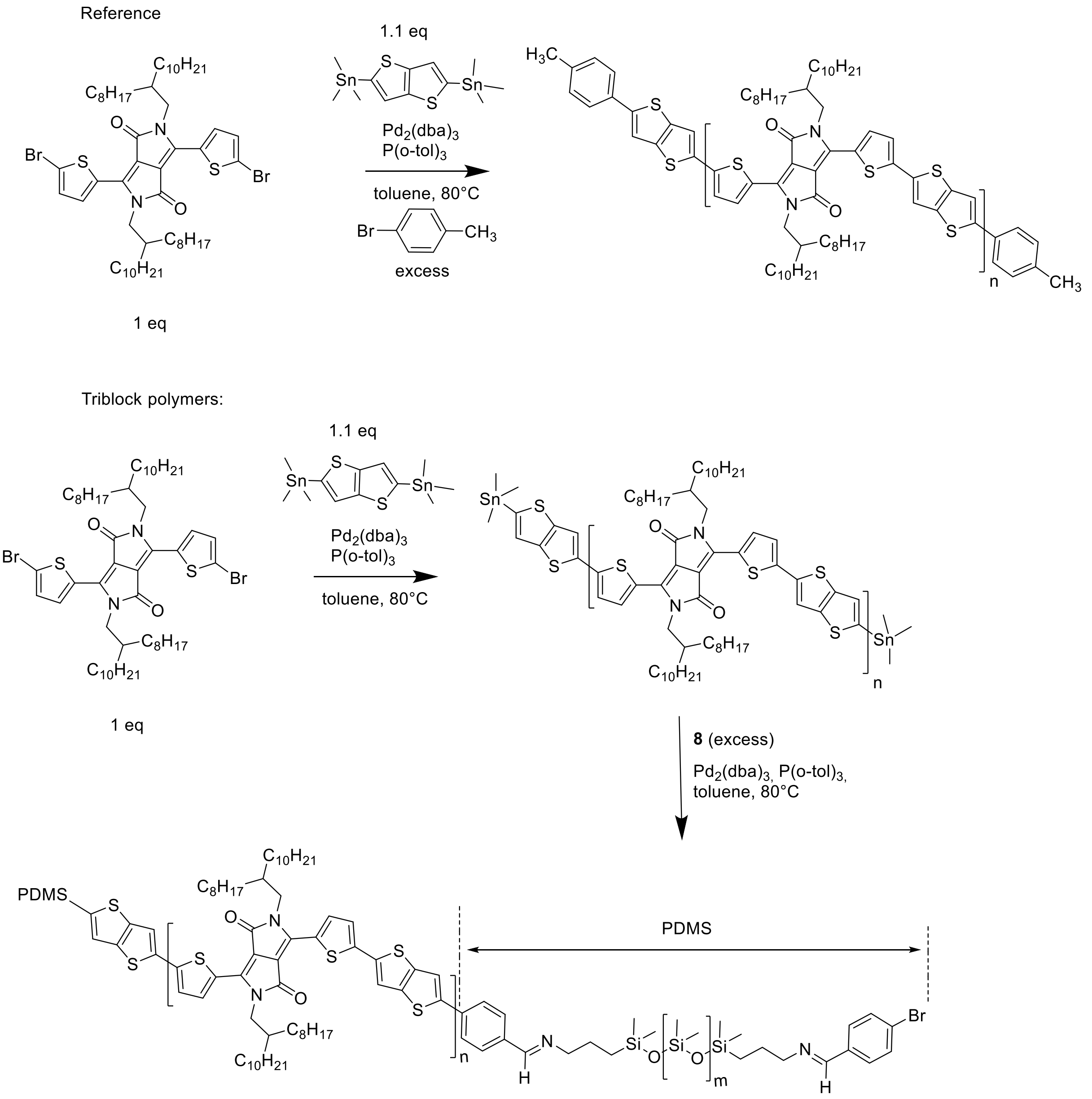

Scheme S 2. Synthesis of the reference polymer and general syntheses route for TBCs.

PDPP-TT polymer (reference polymer)

In a round bottom 2-neck flask, 3,6-bis(5-bromothiophen-2-yl)-2,5-bis(2-octyldodecyl)pyrrolo-[3,4-*c*]pyrrole-1,4(2*H*,5*H*)-dione (153.3 mg, 0.15 mmol, 1.0 eq) (**6**), 2,5-

bis(trimethylstannyl)-thieno[3,2-*b*]thiophene (77.1 mg, 0.16 mmol, 1.1 eq), *tris*(dibenzylidenacetone) dipalladium(0) (5.5 mg, 6 µmol, 0.04 eq) and tri(*o*-tolyl)phosphine (7.32 mg, 24.1 µmol , 0.16 eq) were dissolved in 20 mL degassed anhydrous toluene, and the solution was heated to 80 °C. After stirring for 1 h, 4-bromotoluene (0.75 mmol, 5.0 eq) was added and the stirring continued for another 1 h. The crude reaction mixture was precipitated in MeOH, and the collected solids were purified *via* Soxhlet extraction (MeOH, acetone, hexanes, and DCM). The polymer was isolated by extracting with chloroform and precipitating the chloroform fraction in MeOH, filtration and drying under vacuum (150 mg, 65%).

Triblock polymers (general procedure)

PDPP-TT-PDMS-2.5k

In a round bottom 2-neck flask, 3,6-*bis*(5-bromothiophen-2-yl)-2,5-*bis*(2-octyldodecyl)pyrrolo-[3,4-*c*]pyrrole-1,4(2*H*,5*H*)-dione (153.3 mg, 0.15 mmol, 1.0 eq) (**6**) and 2,5-*bis*(trimethylstannyl)-thieno[3,2-*b*]thiophene (77.1 mg, 0.16 mmol, 1.1 eq), *tris*(dibenzylidenacetone) dipalladium (0) (5.5 mg, 6 µmol, 0.04 eq) and tri(*o*-tolyl)phosphine (7.32 mg, 24.1 µmol , 0.16 eq) were dissolved in 20 mL degassed anhydrous toluene and the solution was heated up to 80 °C. After stirring for 1 hour, (1*E*,1'*E*)-*N*,*N'*-((1,1,3,3-tetramethylpolysiloxane-1,3-diyl)*bis*(propane-3,1-diyl))*bis*(1-(4-bromophenyl)methanimine) (**8**) (molecular weight of 2500 g/mol, 1.2 g, 0.45 mmol, 3.0 eq) was added to the reaction mixture and left to stir for 16 h at 80 °C. Following the precipitation of the crude reaction mixture in MeOH, the collected solids were collected and purified *via* Soxhlet extraction using MeOH, acetone, and hexane. The polymer was collected in DCM, and the fraction precipitated in MeOH and collected *via* centrifugation. The polymer was then dried *in vacuo* and obtained as a rubber-like dark-green solid (275 mg).

PDPP-TT-PDMS-1k and *PDPP-TT-PDMS-25k* were synthesized correspondingly.

2. Characterization Data

Nuclear Magnetic Resonance (NMR) Spectroscopy

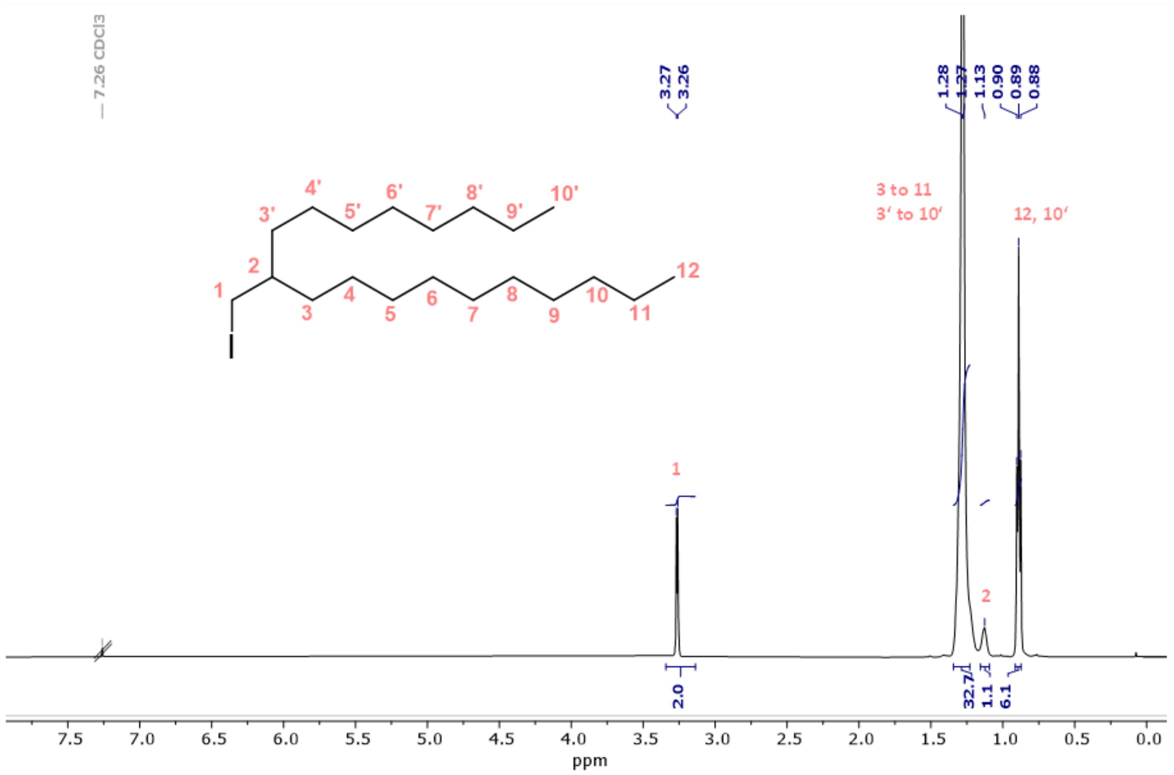

Figure S1. ^{1}H NMR spectra of (**2**) in $CDCl_3$.

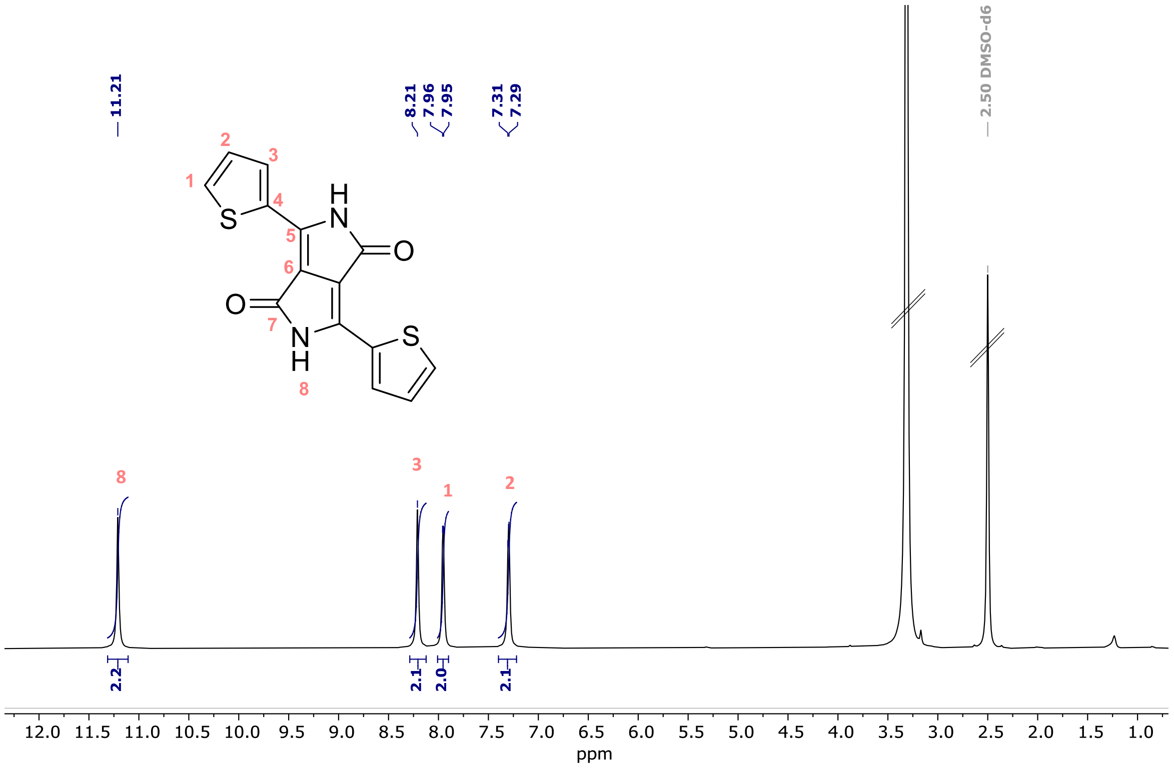

Figure S2. ^{1}H NMR spectrum of (**4**) in DMSO-d_6.

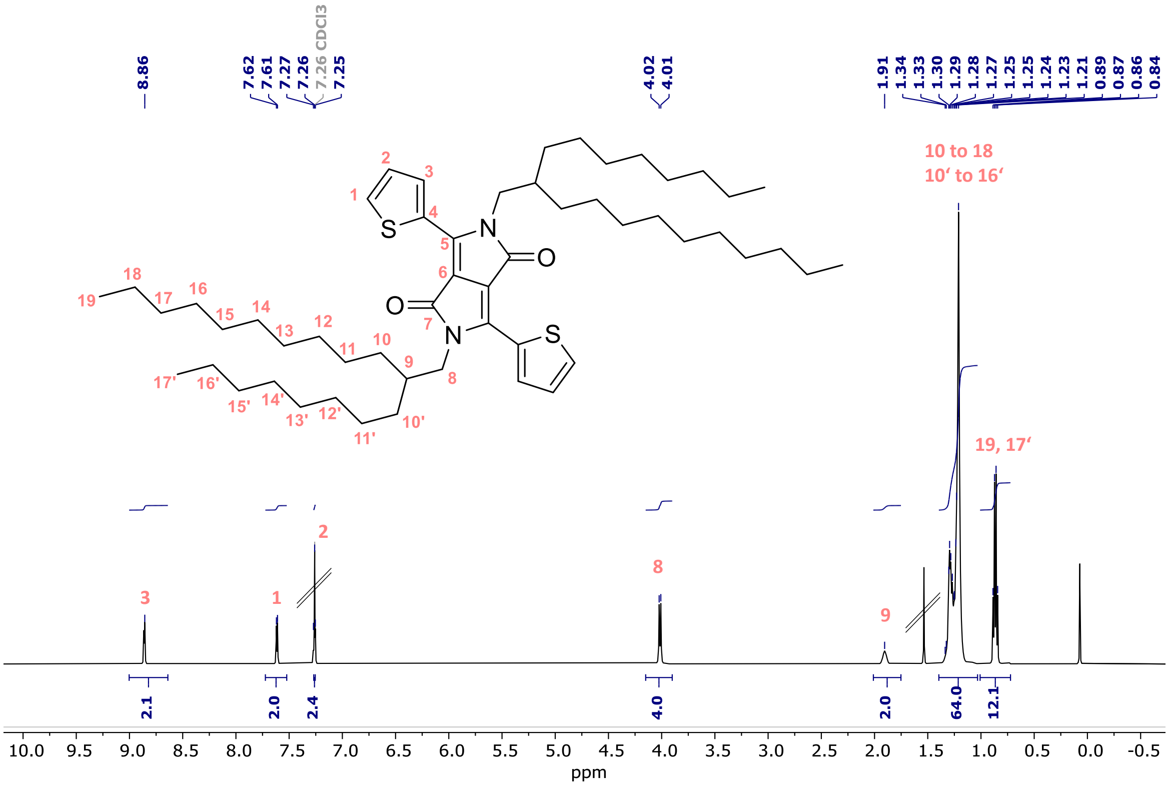

Figure S3. ^{1}H NMR spectrum of (**5**) in $CDCl_3$.

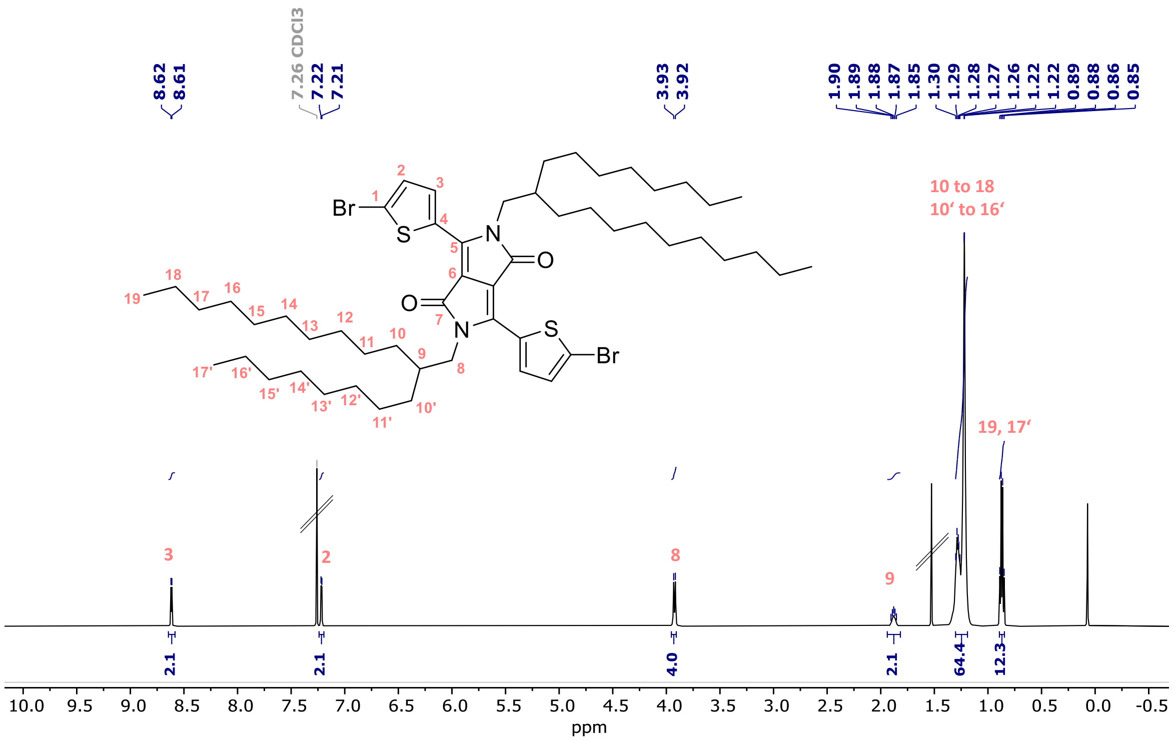

Figure S4. ^{1}H NMR spectrum of (**6**) in $CDCl_3$.

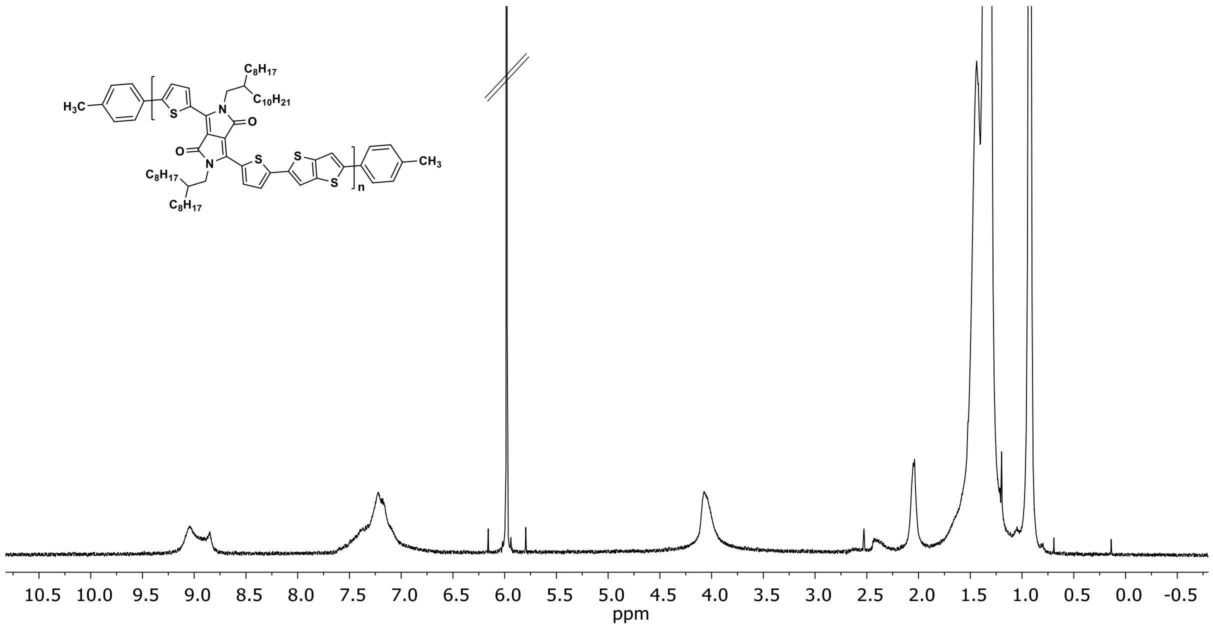

Figure S5. ^{1}H NMR spectrum of PDPP-TT reference polymer in 1,1,2,2-tetrachloroethane-d_2 at 120 °C showing broad and ill-defined signals for the backbone protons due to aggregation.

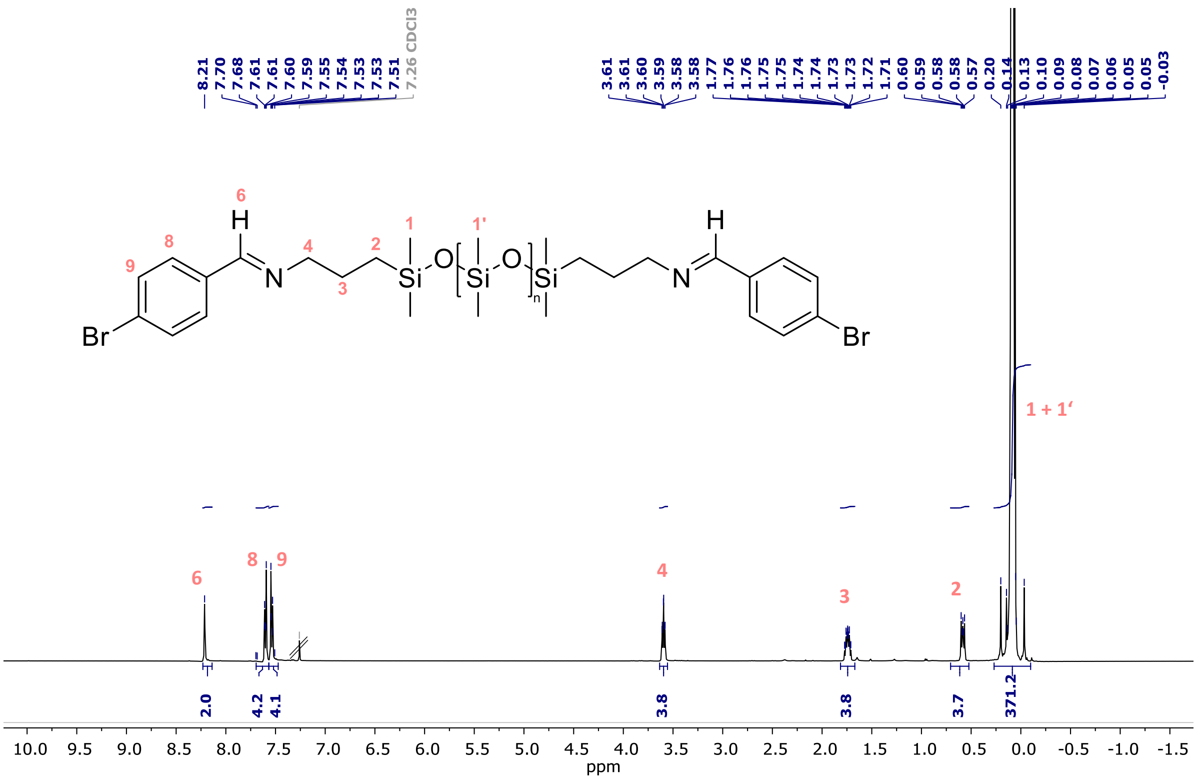

Figure S6. ^{1}H NMR spectrum of (**8**) in $CDCl_3$.

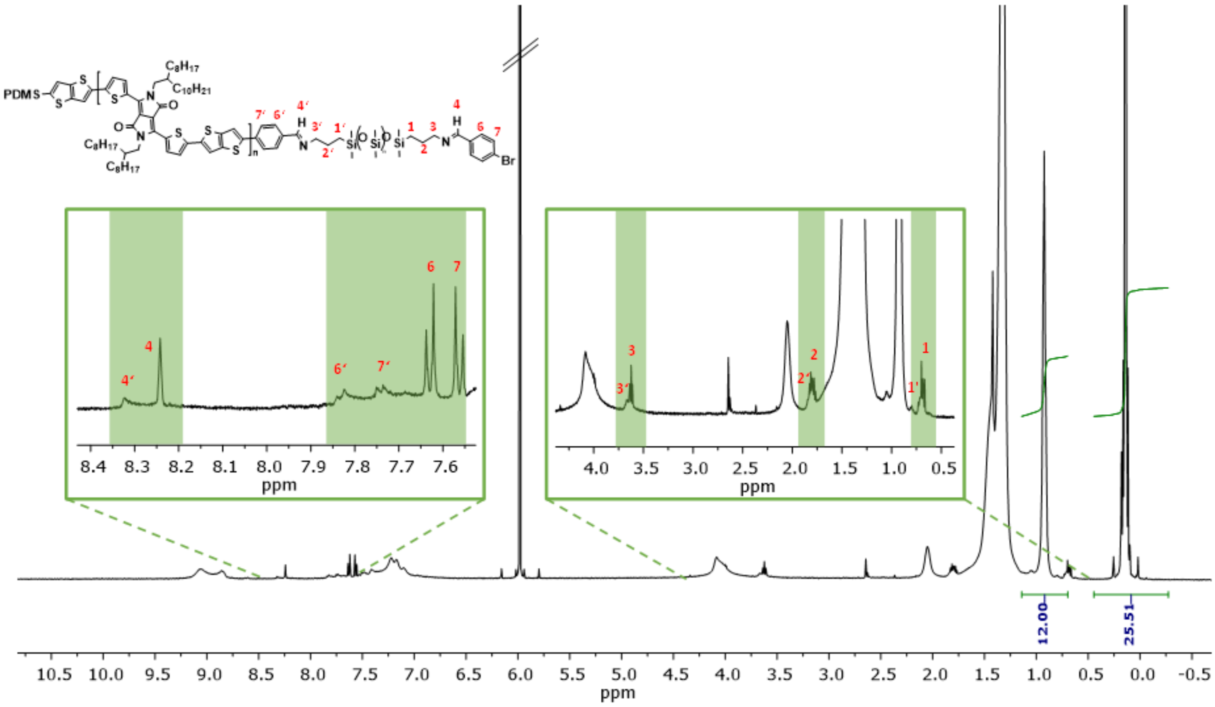

Figure S7. ^{1}H NMR spectrum of PDPP-TT-PDMS-1k block copolymer in 1,1,2,2-tetrachloroethane-d_2 at 120 °C. Inserts highlight the signals of the covalent bond between PDMS block and DPP-TT block ($H_{1'}$ – $H_{7'}$, broadened) and the signals of unreacted PDMS end groups ($H_{1'}$ – $H_{7'}$, narrow).

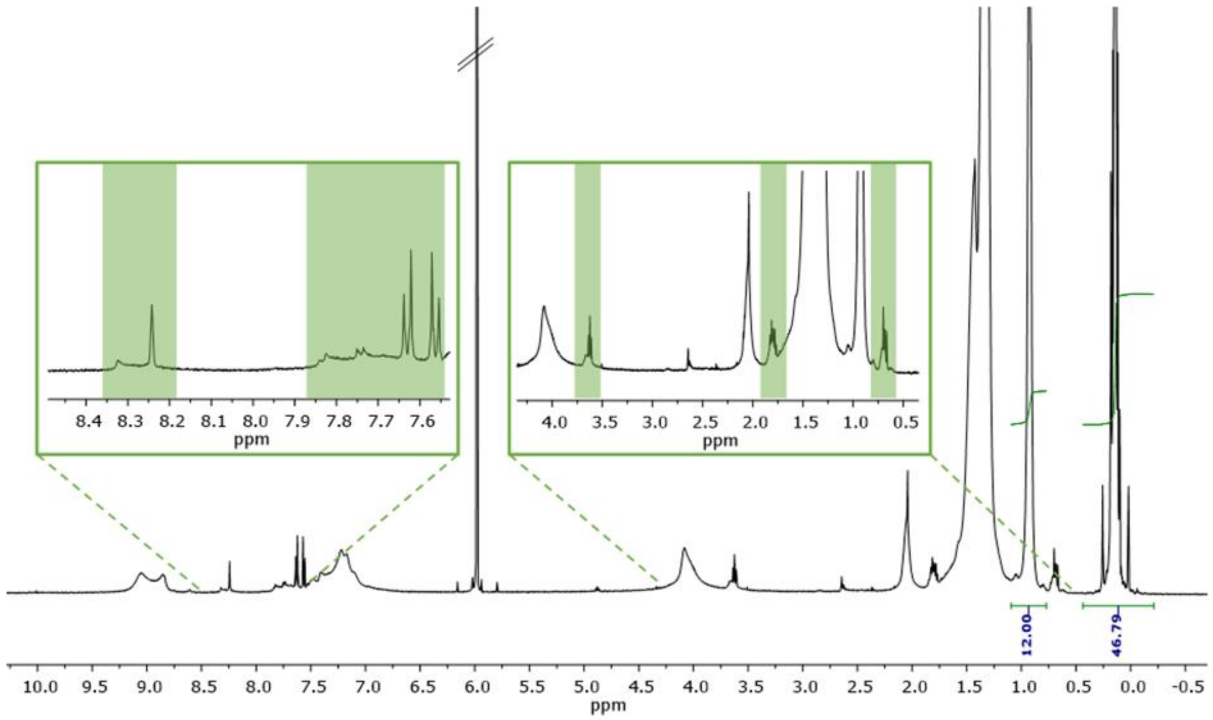

Figure S8. ^{1}H NMR spectrum of PDPP-TT-PDMS-2.5k block copolymer in 1,1,2,2-tetra-chloroethane-d_2 at 120 °C. Inserts are to highlight the signals of the covalent bond between PDMS block and DPP-TT block and the signals of unreacted PDMS end groups. For assignments, see Figure **S7**.

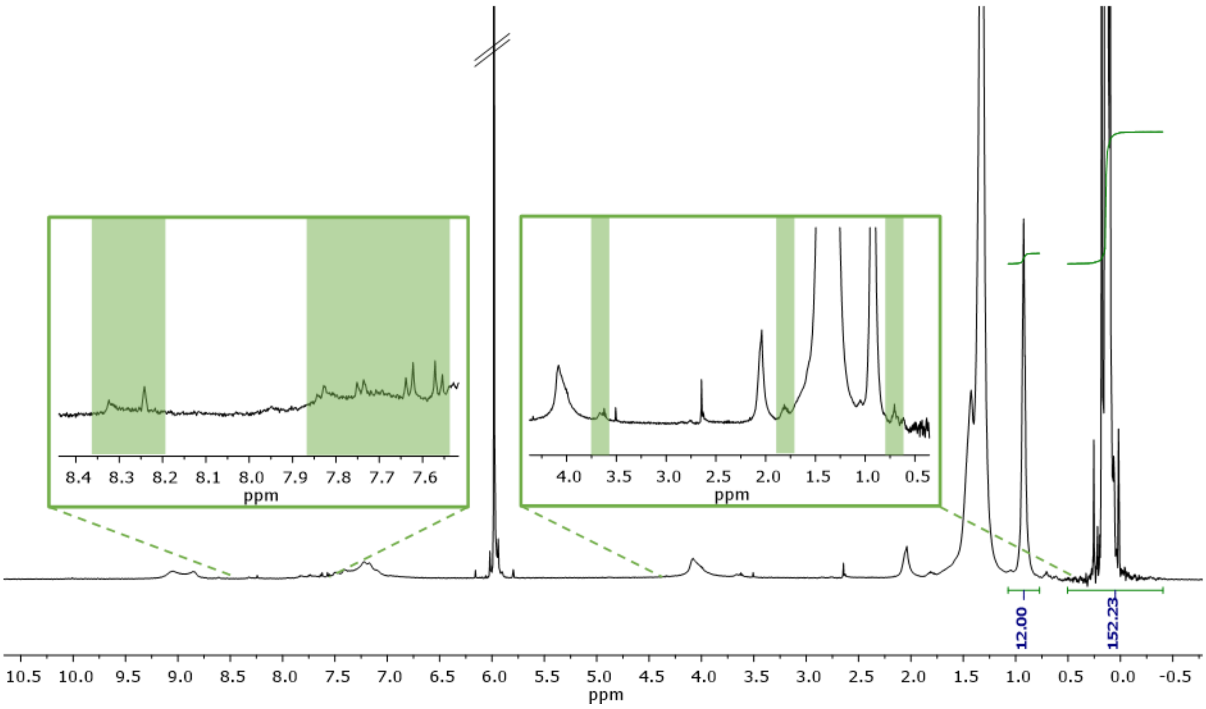

Figure S9. ^{1}H NMR spectra of PDPP-TT-PDMS-25k block copolymer in 1,1,2,2-tetra-chloroethane-d_2 at 120 °C. Inserts are to highlight the signals of the covalent bond between PDMS block and DPP-TT block and the signals of unreacted PDMS end groups. For assignments, see Figure **S7**.

Differential Scanning Calorimetry (DSC)

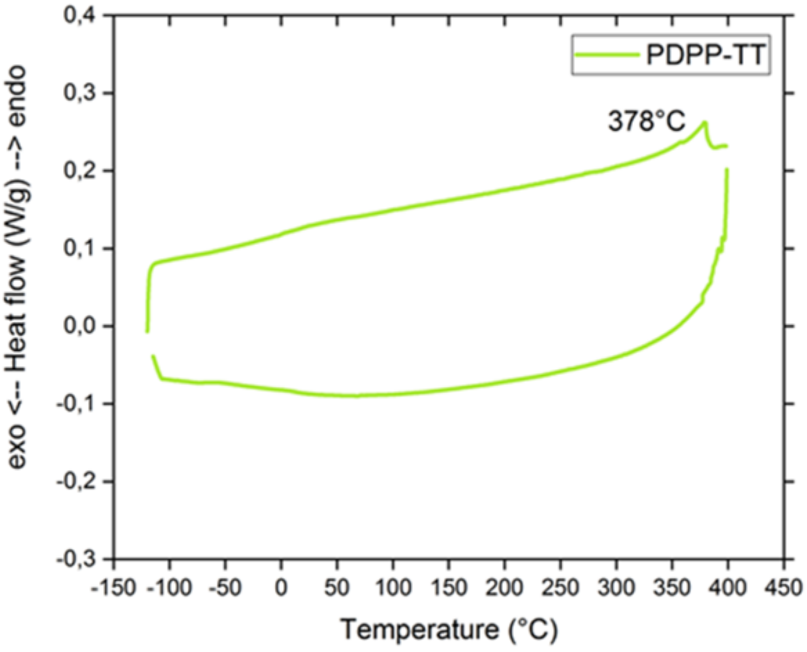

Figure S10. DSC curve of PDPP-TT neat polymer. The heating rate was 10 K / min.

Table S1. Molecular weights and chemical composition of the TBCs and the reference PDPP-TT: M_w [g/mol], M_n [g/mol] and weight dispersity (PDI) as determined by HT GPC using a PS calibration standard, number of repeating units of PDMS calculated based on M_n [g/mol] of the starting materials, and PDMS content in wt.-% as calculated from the HT ^{1}H NMR spectra.

Polymer	M_w [g/mol] /PDI	M_n [g/mol]	PDMS: number of repeating units (theoretical) based on M_n	PDMS wt.- % content
**PDPP-TT**	65700/3.71	17700	0	0
**PDPP-TT-1000**	50300/3.59	14000	12	24
**PDPP-TT-2500**	48900/3.35	14600	33	37
**PDPP-TT-25000**	95000/2.53	37500	330	65

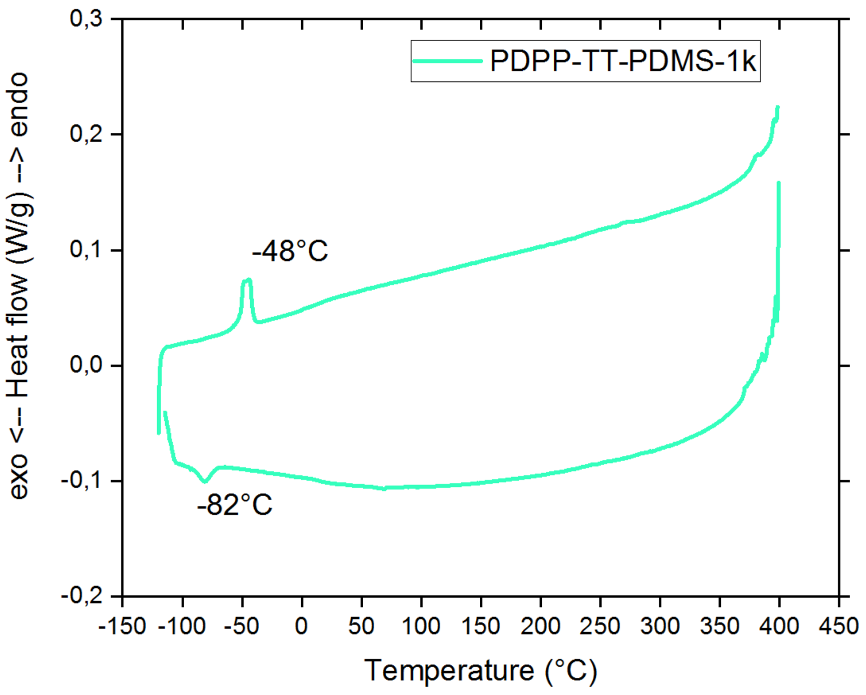

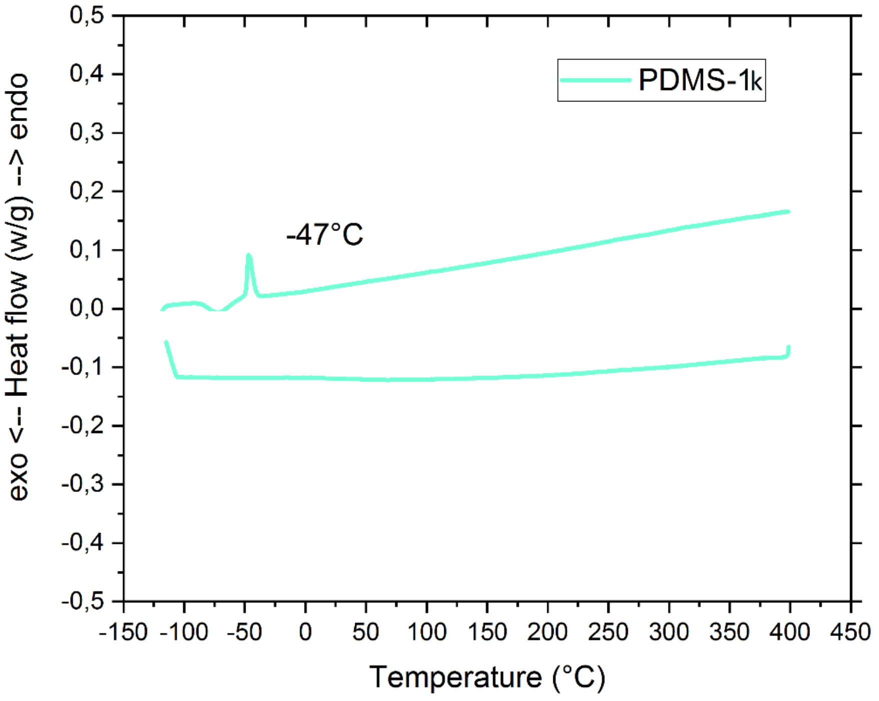

Figure S11. DSC curve of PDPP-TT-PDMS-1000 (top) and modified PDMS-1000 (bottom). The heating rate was 10 K/min.

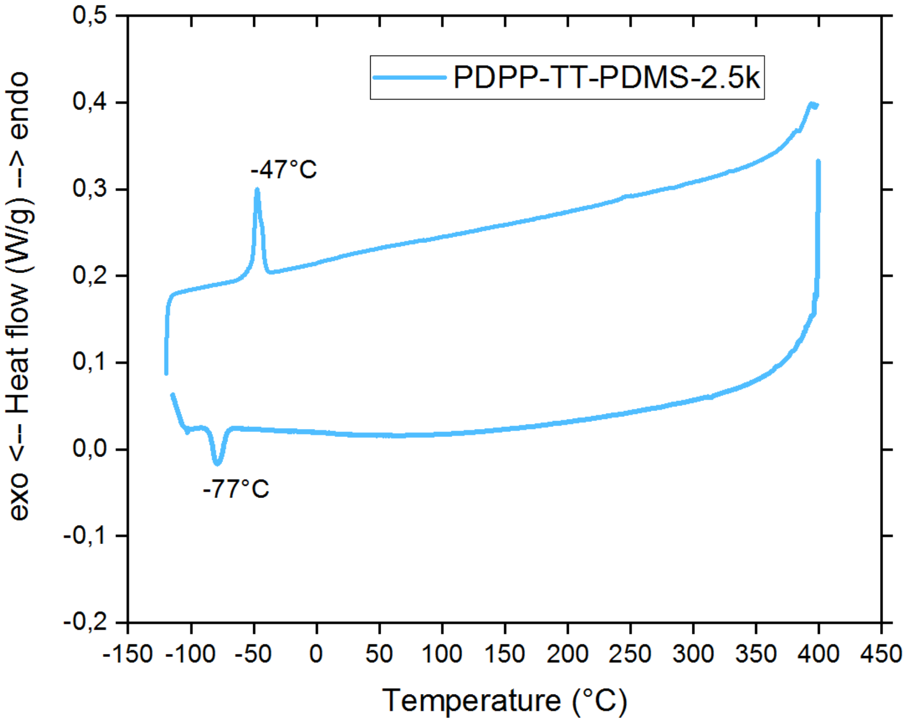

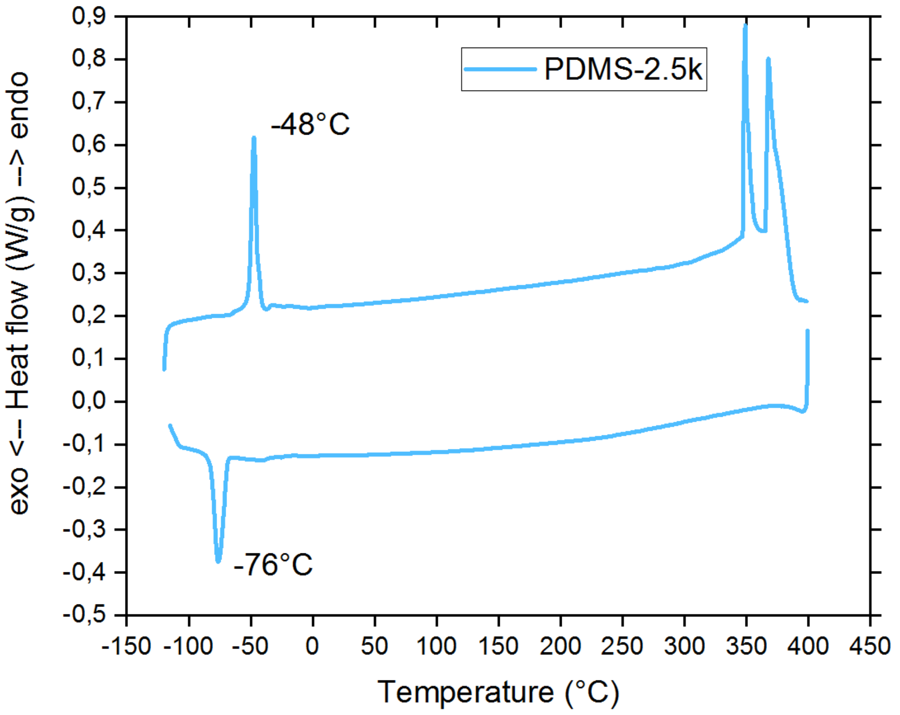

Figure S12. DSC curve of PDPP-TT-PDMS-2.5k (top) and modified PDMS-2.5k (bottom). The heating rate was 10 K/min.

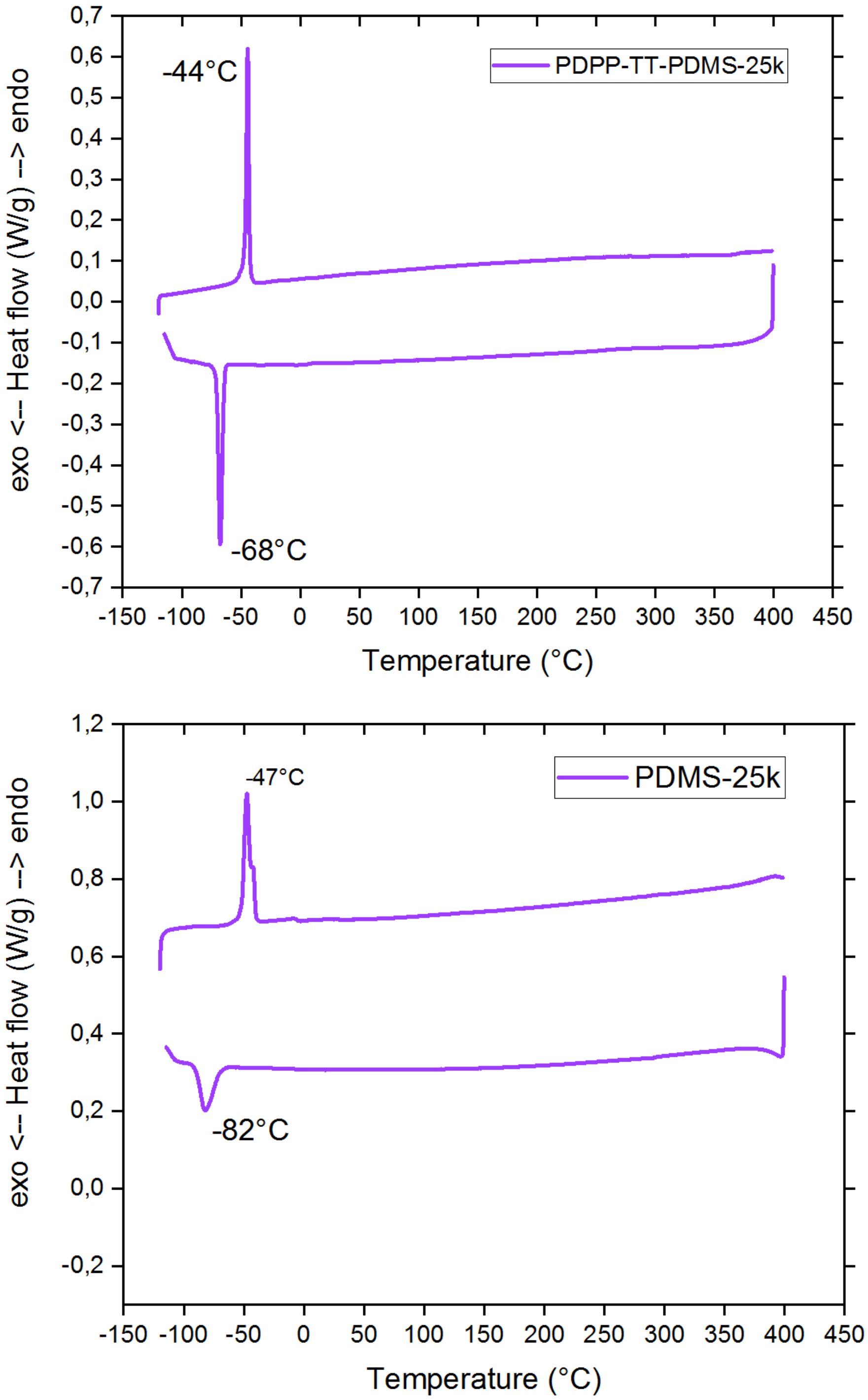

Figure S13. DSC *c*urve of PDPP-TT-PDMS-25k (top) and modified PDMS-25k (bottom). The heating rate was 10 K/min.

Thermogravimetric Analysis (TGA)

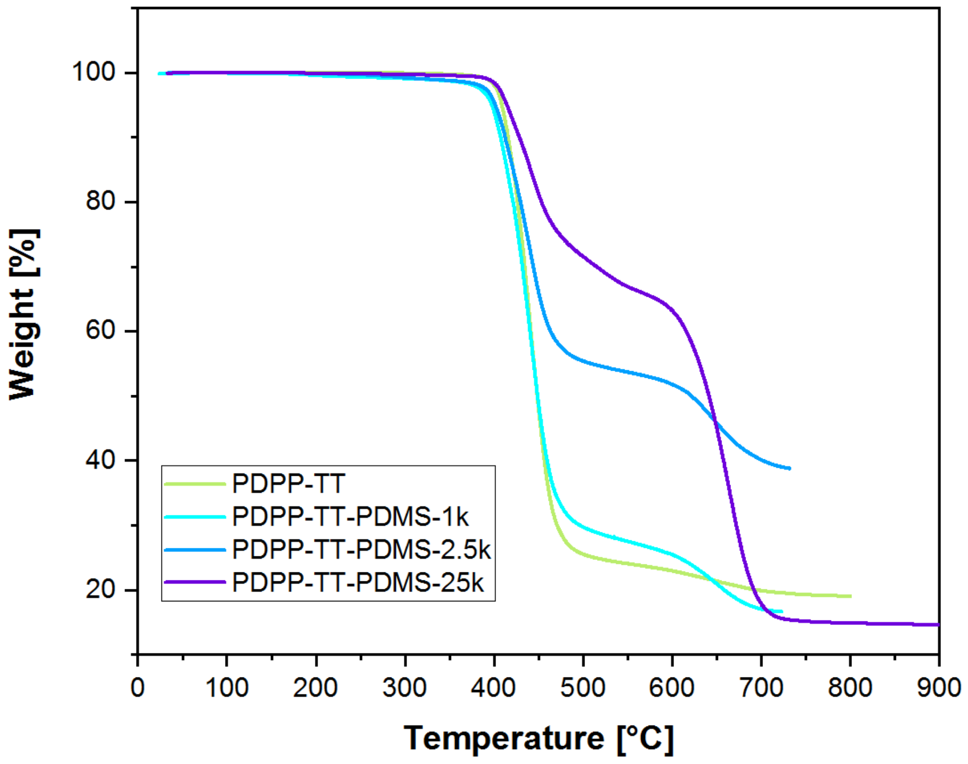

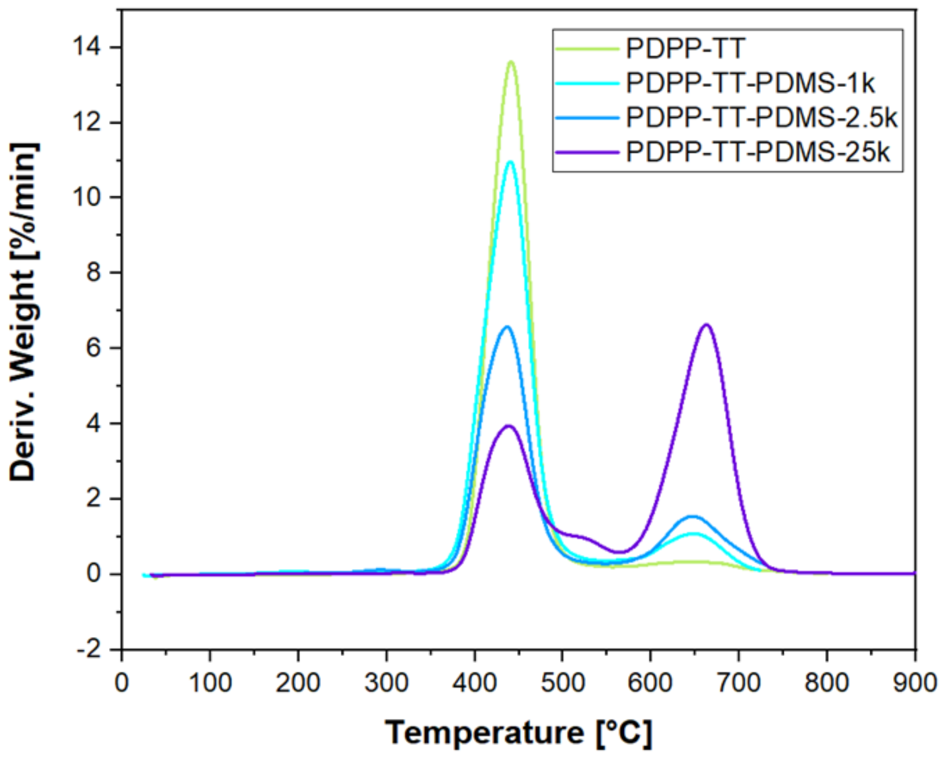

Figure S14. TGA curves of the reference PDPP-TT and block copolymers.

Table S2. Results of TGA analysis for the reference PDPP-TT and block copolymers. TGA analysis was carried out under nitrogen atmosphere with the isothermal for 30 min and ramp 10.00 K/min to 1000 °C. T_{min} of derivative curve refers to the end of each decomposition step.

Sample name	Weight (mg)	Weight loss after 30' iso at RT (%)	T_{max} of derivative curve (°C)	T_{min} of derivative curve (°C)	Weight loss at T_{min} (%)	Residue at 800°C (%)	Residue at 1000°C (%)	Residue after switching to air[a] (%)
**PDPP-TT**	3.541	0	-<br>441<br>641	~370<br>550<br>to 800	0.4<br>75.6<br>5.0	-	-	19.0
**PDPP-TT-PDMS-1k**	4.155	0	190<br>440<br>650	255<br>550<br>760	0.6<br>71.9<br>11.3	16.3	15.3	2.7
**PDPP-TT-PDMS-2.5k**	6.519	0	291<br>435<br>647	330<br>550<br>to 800	1.0<br>45.2<br>15.7	38.0	37.2	17.6
**PDPP-TT-PDMS-25k**	6.305	0	438/s525<br>663	565<br>840	33.9<br>51.4	14.9	13.9	4.4

[a] For these measurements, a heating scan (isothermal for 30 min and ramp 10.00 K/min to 1000 °C) under nitrogen was carried out, then the N_2 supply was switched off and an additional isothermal scan was run under air.

Ultraviolet-visible (UV/Vis) Spectroscopy

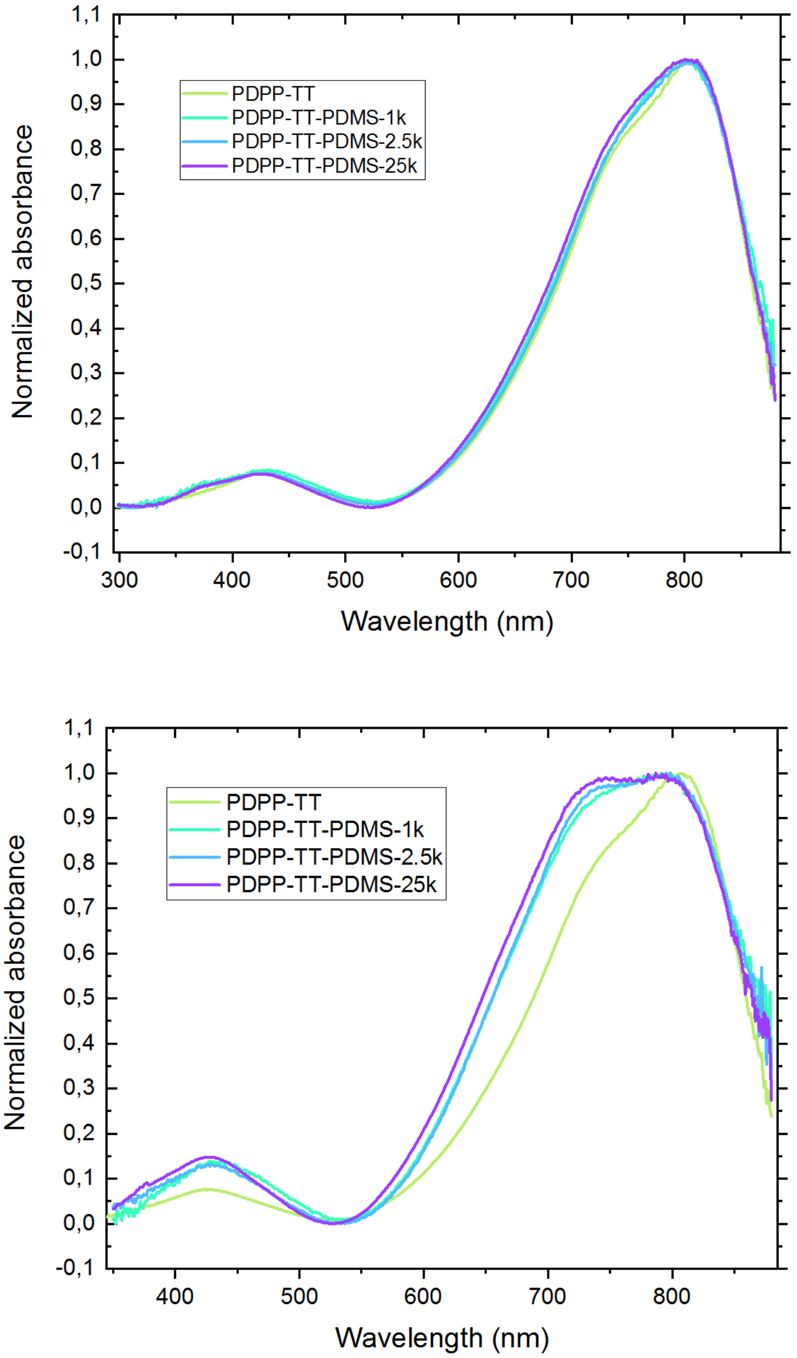

Figure S15. UV-Vis absorption spectra of PDPP-TT, PDPP-TT-PDMS-1000, PDPP-TT-PDMS-2500 and PDPP-TT-PDMS-25000 polymers in a ~ 1 mg/mL chlorobenzene solution (top) and spin-coated from a ~ 5 mg/mL solution (bottom).

Cyclic Voltammetry (CV)

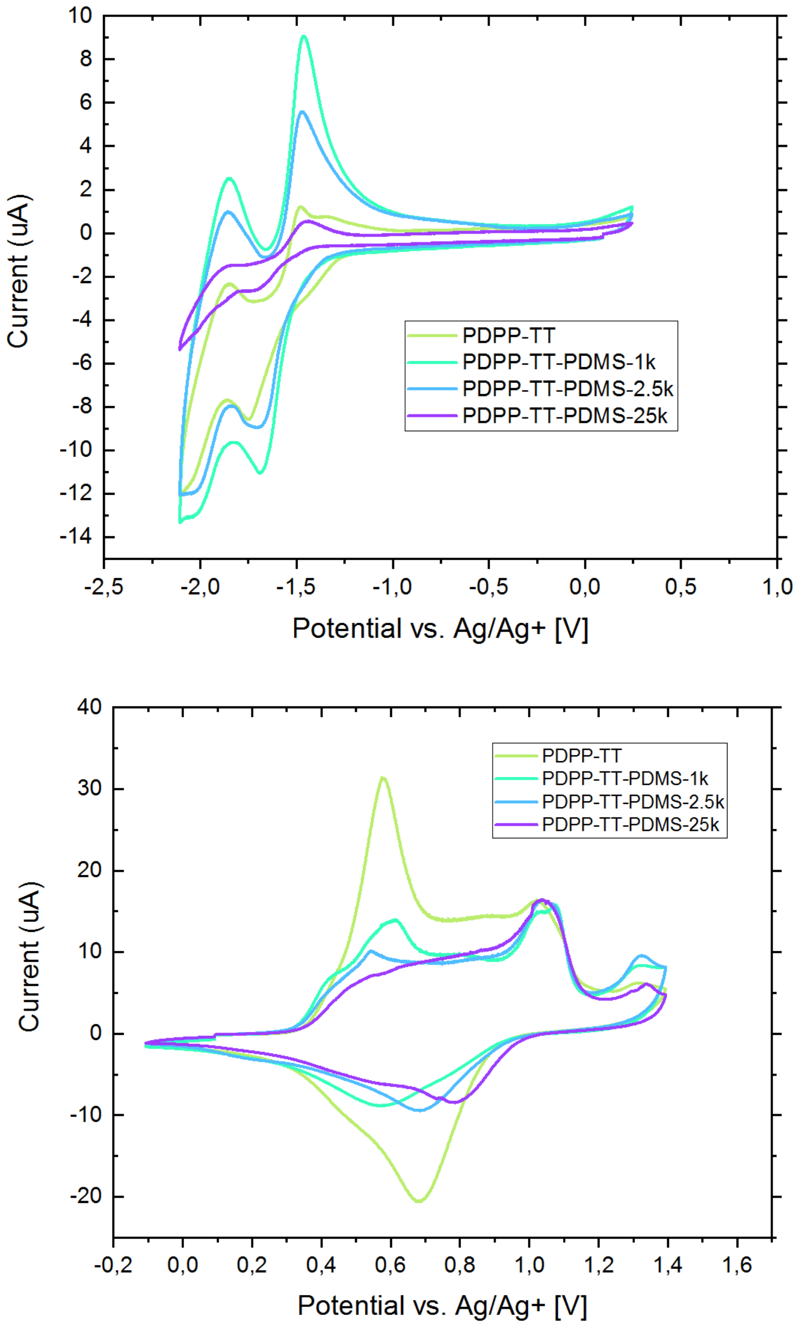

Figure S16. Cyclic voltammograms[b] showing reduction (top) and oxidation (bottom) sections for thin films of PDPP-TT, PDPP-TT-PDMS-1k, PDPP-TT-PDMS-2.5k and PDPP-TT-PDMS-25k.

[b] the reference and the TBCs exhibit similar electrochemically irreversible behavior. The shape of the voltammograms depends on the film quality rather than on the location of the oxidation and reduction onset potentials.

Table S3. Summary of the reduction and oxidation onsets of the polymers PDPP-TT, PDPP-TT-PDMS-1000, PDPP-TT-PDMS-2500 and PDPP-TT-PDMS-25000 as well as their respective energy levels calculated with Eq. **(1)** and Eq. **(2)**, UV/Vis absorption and calculated optical band gap.

Polymer name	$E_{onset, ox}$ (V)	$E_{onset, red}$ (V)	E_{HOMO} (eV)	E_{LUMO} (eV)	λ_{max}^{abs} [nm]		E_g^{opt} [eV]
					solution	film	
**PDPP-TT**	0.34	-1.27	-5.32	-3.72	803		1.6
**PDPP-TT-PDMS-1k**	0.32	-1.48	-5.30	-3.50	803		1.8
**PDPP-TT-PDMS-2.5k**	0.33	-1.50	-5.31	-3.48	803		1.83
**PDPP-TT-PDMS-25k**	0.35	-1.49	-5.33	-3.49	803		1.84

Grazing Incidence Wide Angle X-ray Scattering (GIWAXS)

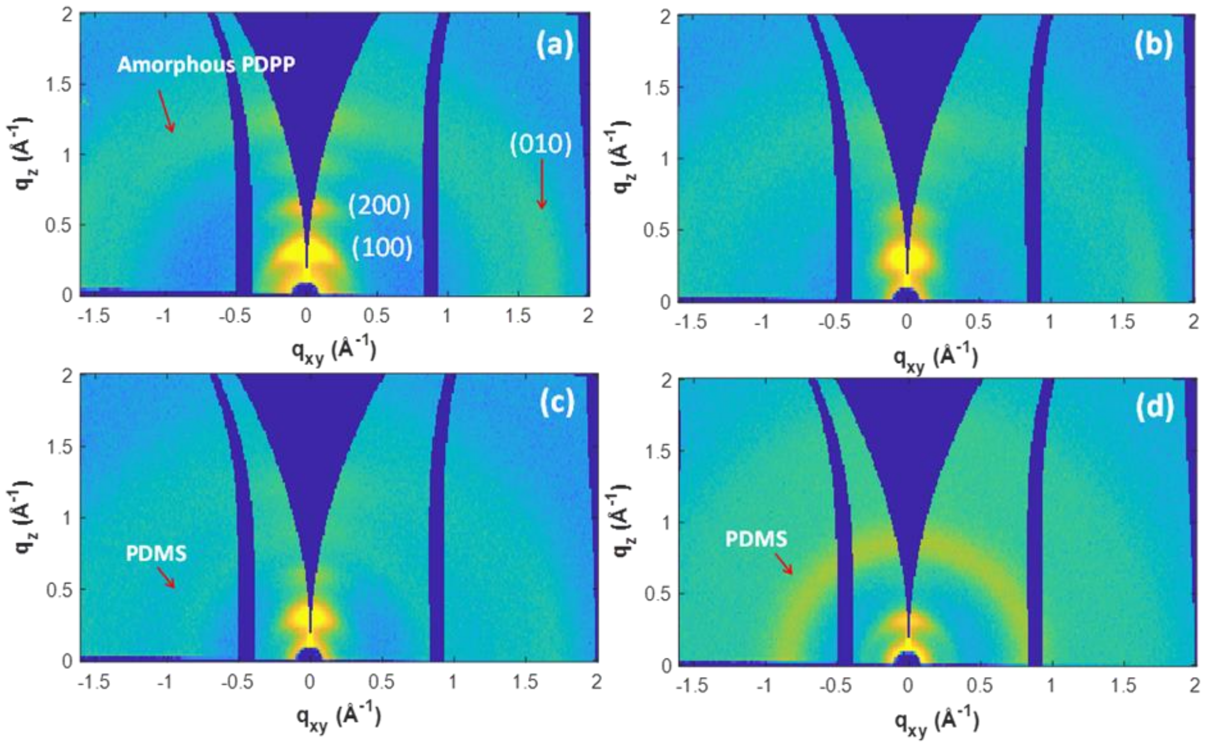

Figure S17. GIWAXS patterns for spin-coated films of (a) PDPP-TT, (b) PDPP-TT-PDMS-1k, (c) PDPP-TT-PDMS-2.5k, (d) PDPP-TT-PDMS-25k. The arrows indicate the amorphous and PDMS signals.

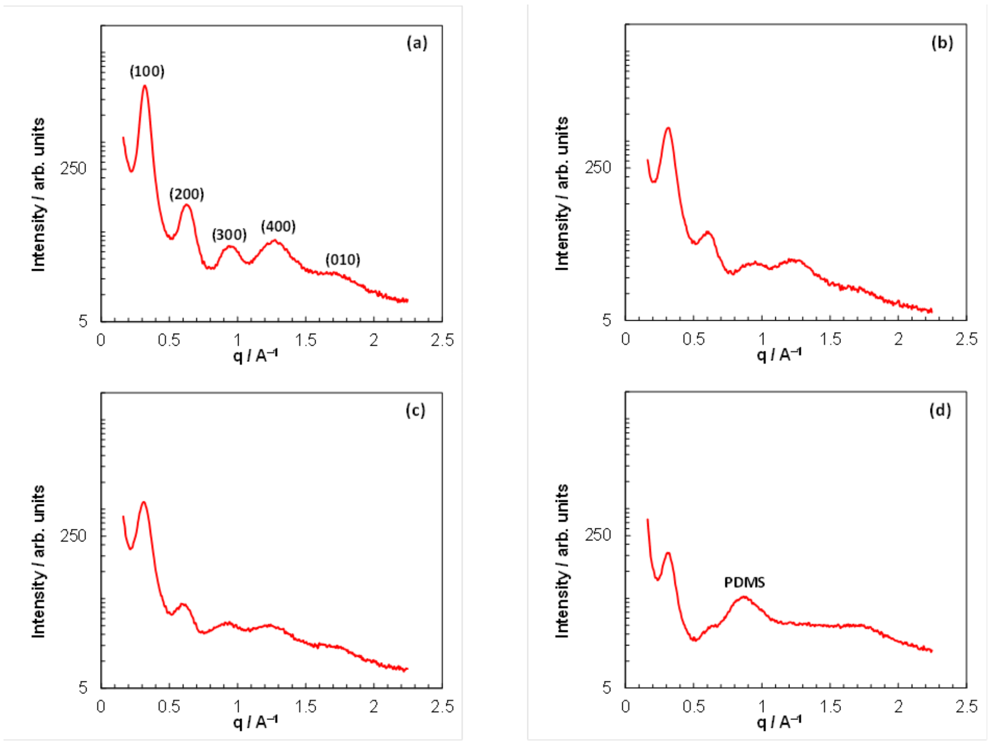

Figure S18. Out-of-plane line cut profiles of the polymer films. (a) PDDP-TT homopolymer, (b) PDPP-TT-PDMS-1k, (c) PDPP-TT-PDMS-2.5k, (d) PDPP-TT-PDMS-25k.

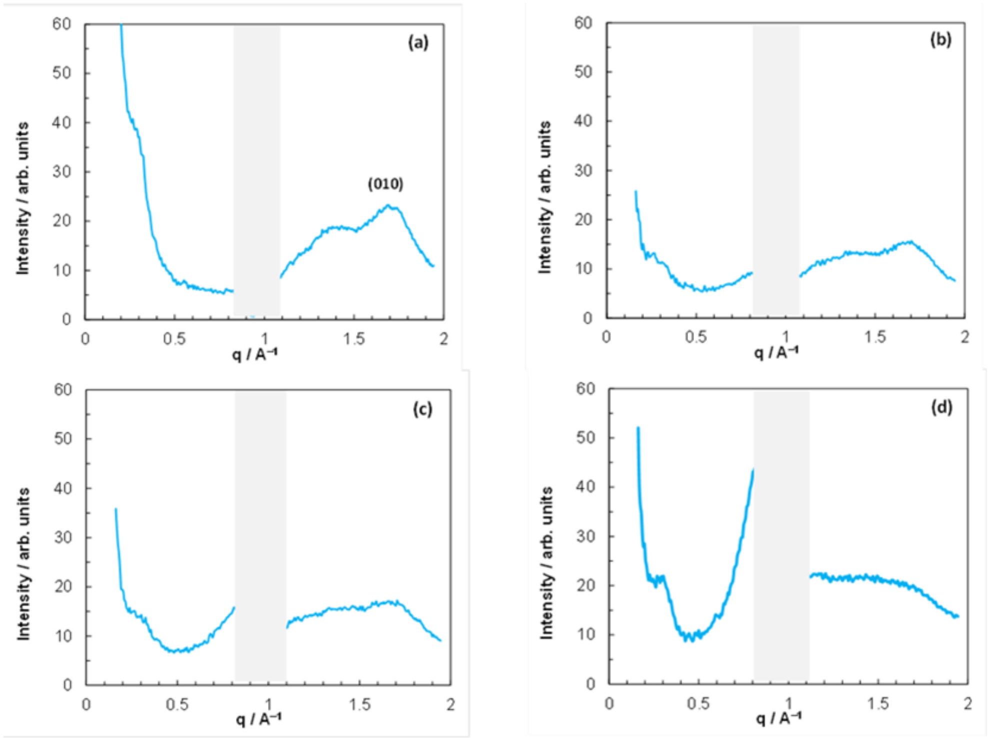

Figure S19. In-plane line cut profiles of the polymer films. (a) PDDP-TT homopolymer, (b) PDPP-TT-PDMS-1k, (c) PDPP-TT-PDMS-2.5k, (d) PDPP-TT-PDMS-25k.

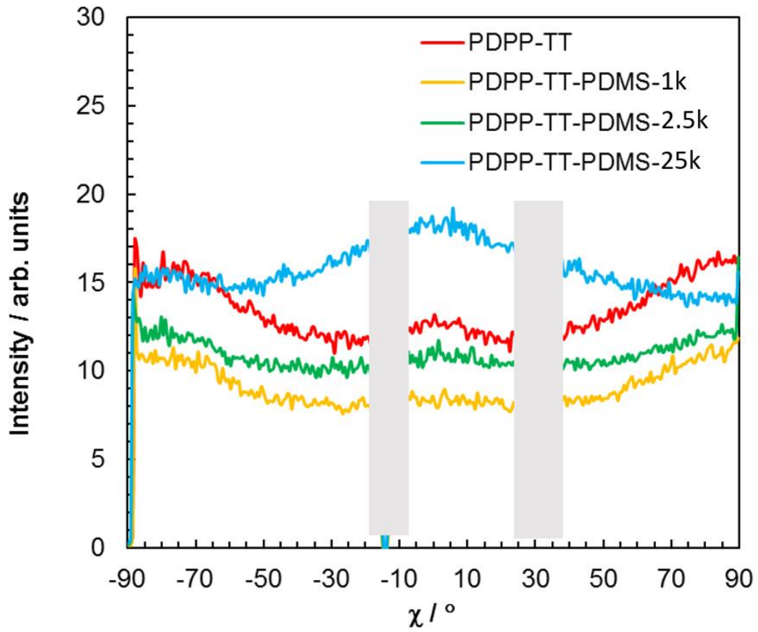

Figure S20. Azimuthal integration profiles of the (010) reflection for PDDP-TT homopolymer, PDPP-TT-PDMS-1k, PDPP-TT-PDMS-2.5k and PDPP-TT-PDMS-25k films.

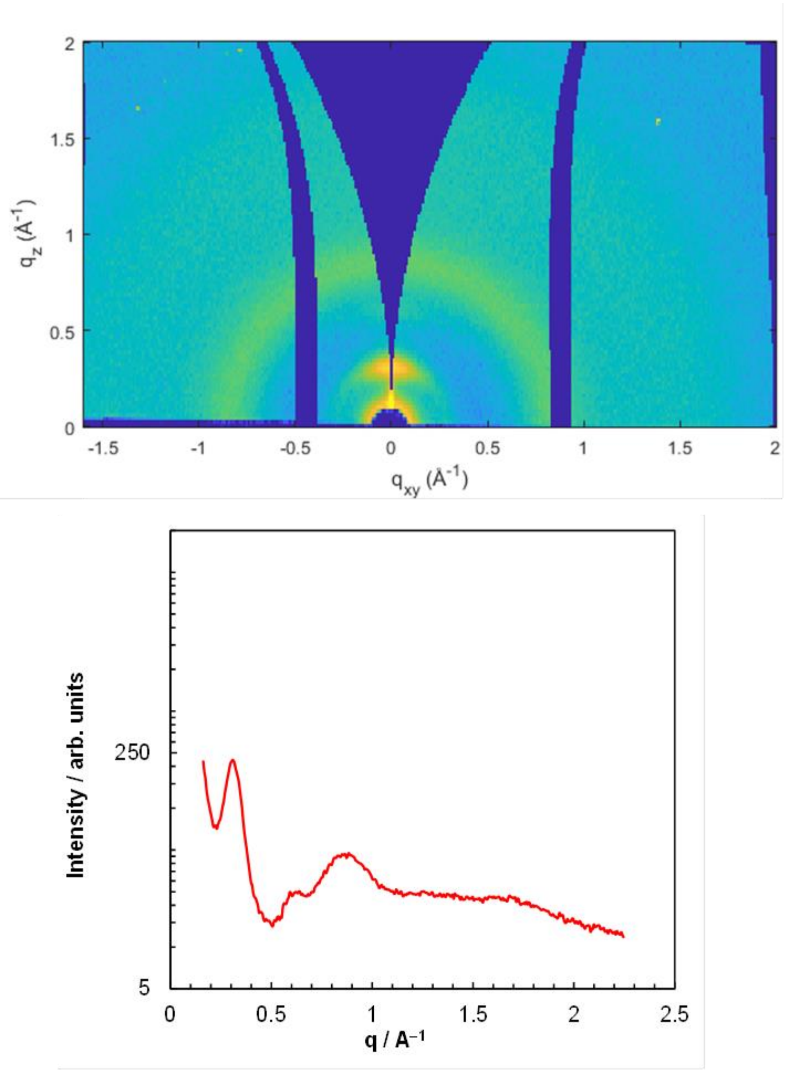

Figure S21. GIWAXS pattern of annealed PDPP-TT-PDMS-25k film (top) and the corresponding out-of-plane line cut profile (bottom).

Table S4. The relative degree of crystallinity, χ, lamellar spacing, d_{100}, and out-of-plane π-π stacking distance, d_{010} of the PDPP-TT block in all investigated polymer films derived from the GIWAXS data.

**Film**	**χ (%)**	$\boldsymbol{d_{100}}$ **(Å)**	$\boldsymbol{d_{010}}$**(Å)**
**PDPP-TT**	84.5	19.72	3.69
**PDPP-TT-PDMS-1k**	71.5	20.50	3.63
**PDPP-TT-PDMS-2.5k**	61.4	20.47	3.70
**PDPP-TT-PDMS-25k**	13.5	20.74	3.65
**PDPP-TT-PDMS-25k (Annealed)**	13.7	20.65	3.67

Atomic Force Microscopy (AFM)

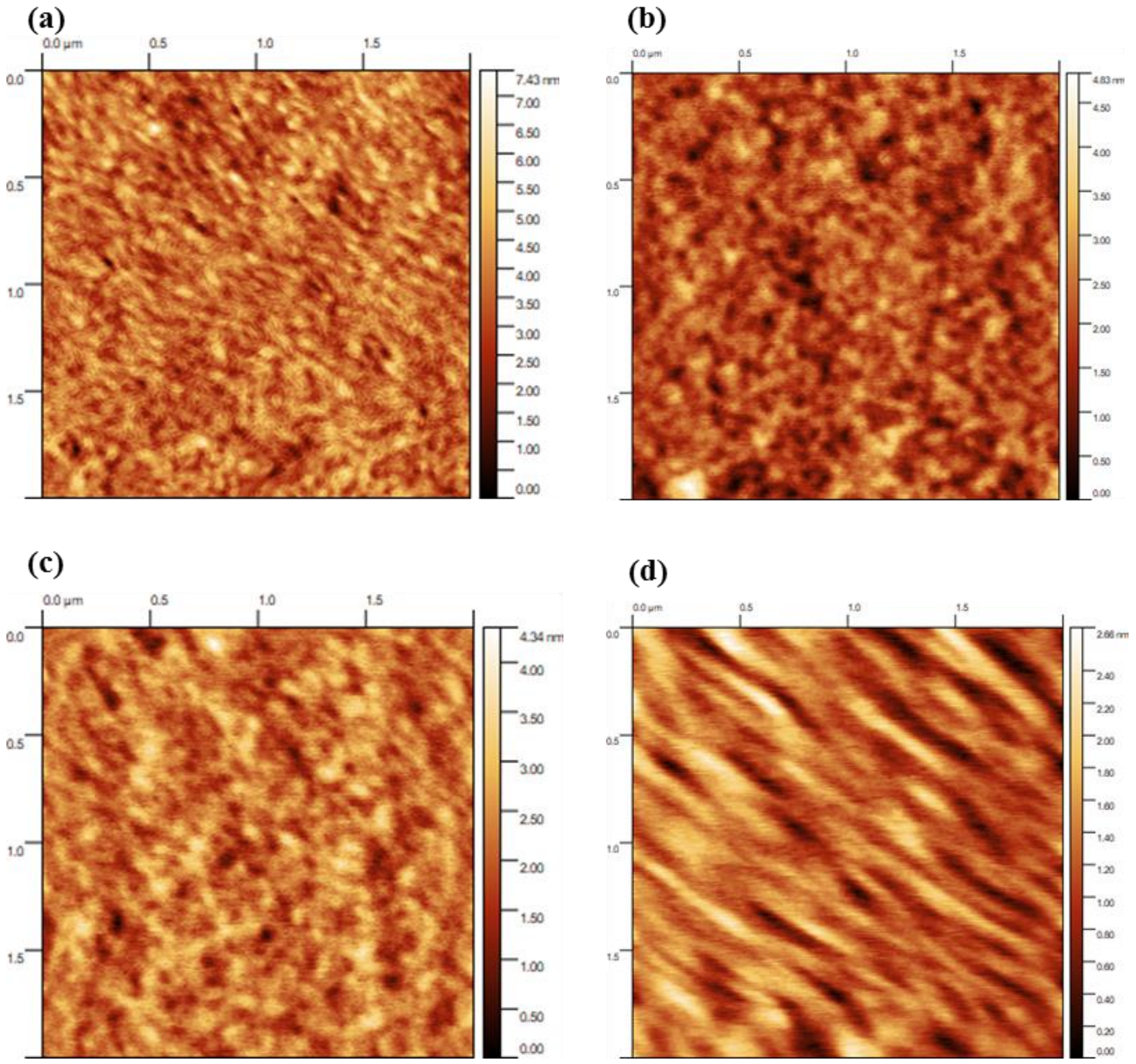

Figure S22. AFM height images of solution sheared samples: (a) PDPP-TT reference, (b) PDPP-TT-PDMS-1k, (c) PDPP-TT-PDMS-2.5k, (d) PDPP-TT-PDMS-25k.

Transmission Electron Microscopy (TEM)

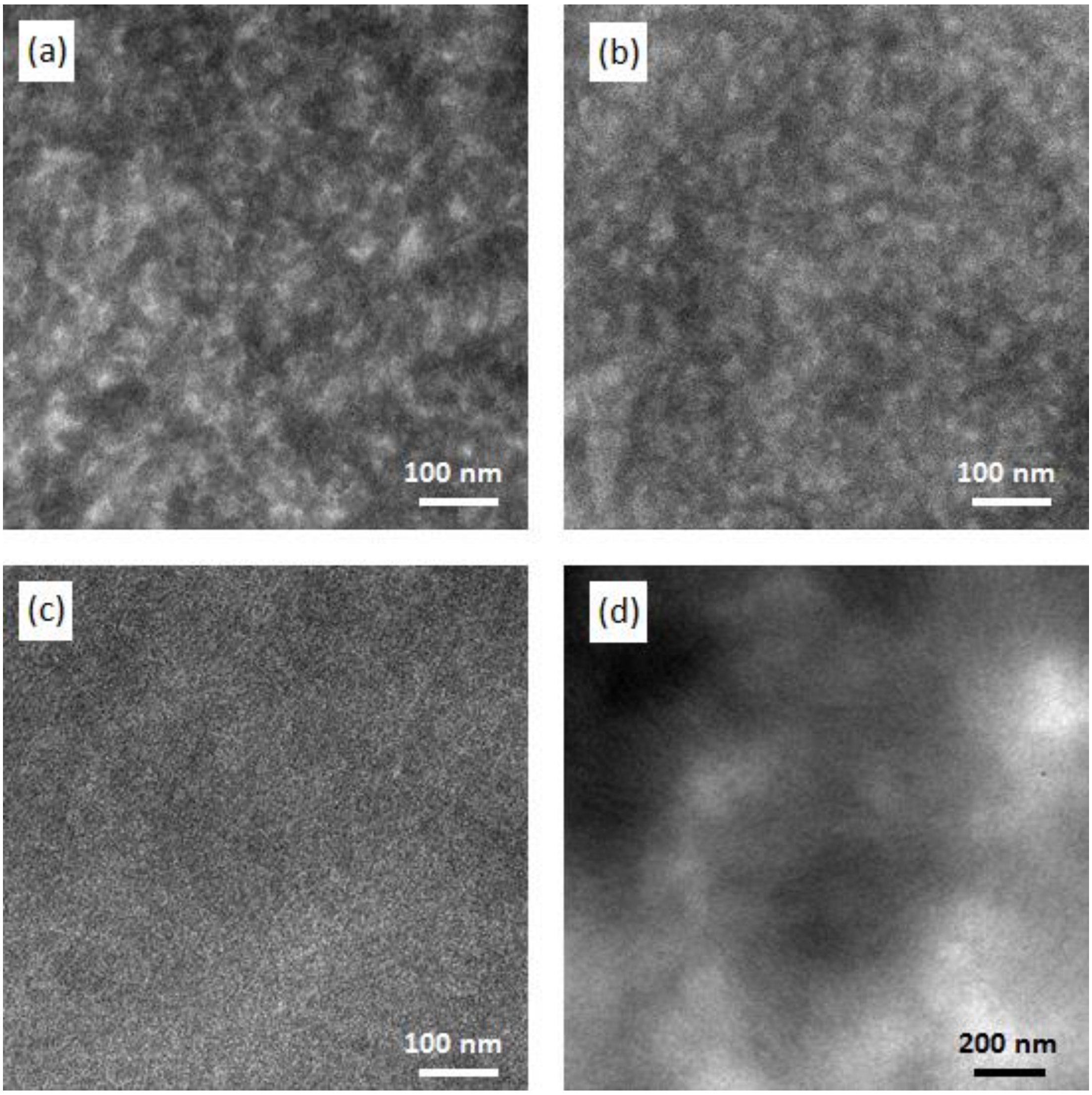

Figure S23. TEM images of (a) PDPP-TT-PDMS-25k, (b) PDPP-TT-PDMS-2.5k, (c) PDPP-TT-PDMS-1k thin films stained with RuO_4 for 5 min and (d) an unstained PDPP-TT thin film. The contrast in (a,b,c) corresponds to nanophase separation in TBC. The large area contrast in (d) correspond to film thickness variation.

Microscopy Under Strain

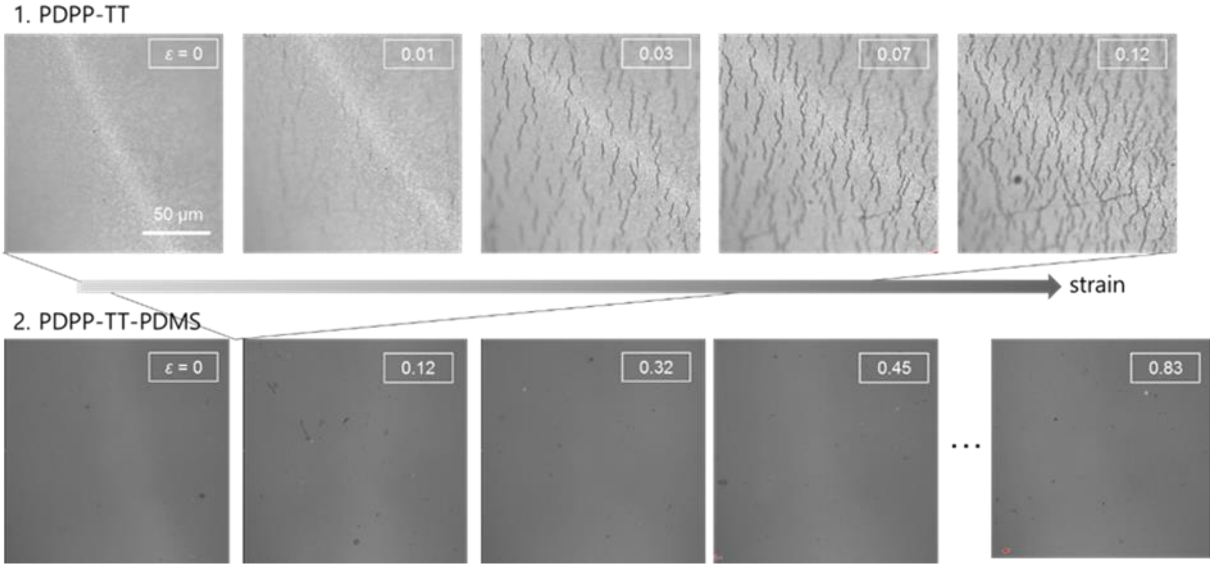

Figure S24. Optical microscope images showing the crack initiation strain of PDPP-TT (top) and PDPP-TT-PDMS-25k (bottom) polymer films on PDMS substrate. Note the difference in total strain.

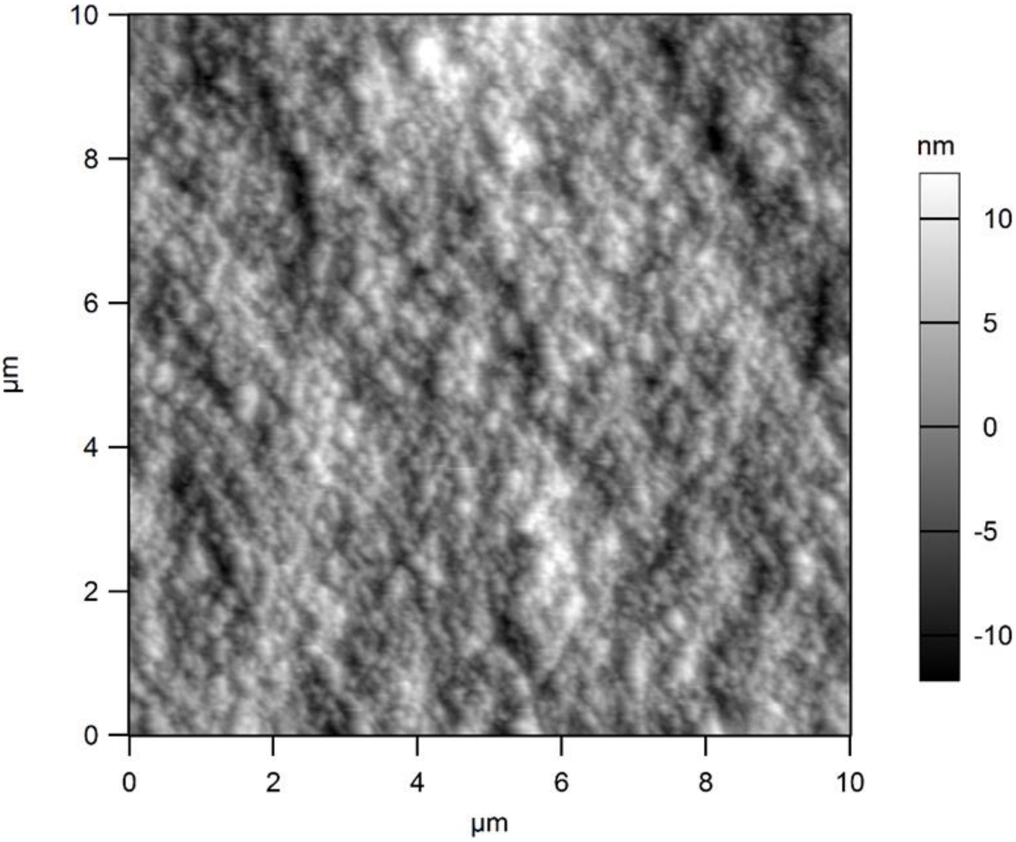

Figure S25. AFM topography of the PDPP-TT-PDMS-25k films at 80 % (ε = 0.8).

3. Electrical Characterization

BGTC and BGBC Transfer Characteristics

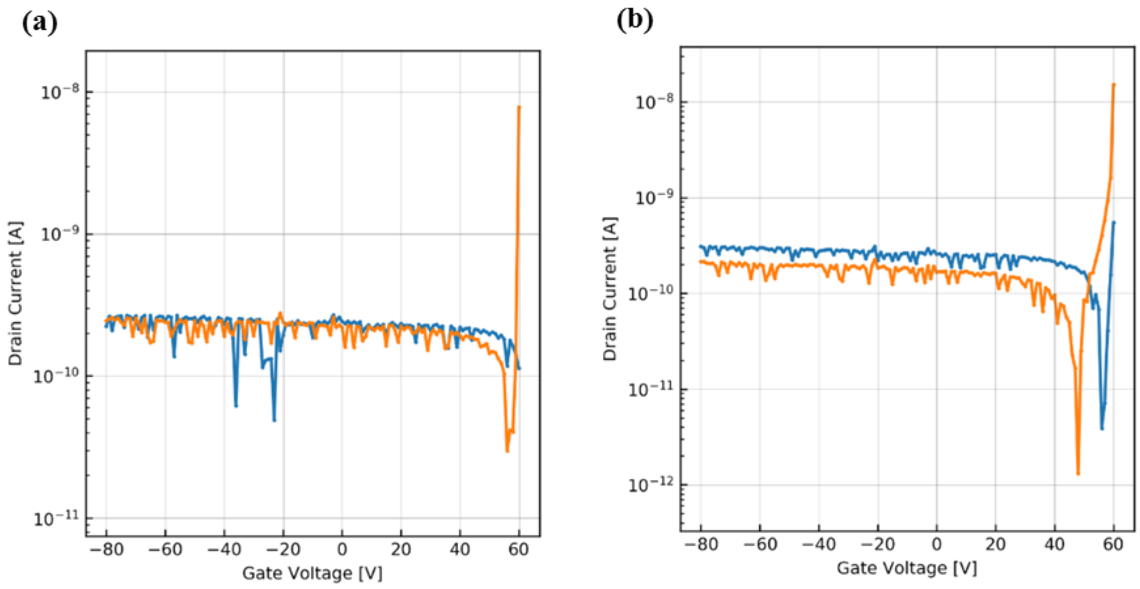

Figure S26. Transfer characteristics of BGBC OFETs of PDPP-TT and PDMS physical blends: (a) 60% PDPP-TT and 40% PDMS (Mw = 2500 g/mol); (b) 25% PDPP-TT and 75% PDMS (Mw = 25000 g/mol).

Table S5. Parameters of BGTC OFETs based on all polymers sheared at 1 mm/s shearing speed.

**Sample name**	**Shearing 1 mm/s**				
	**Mob. Fwd [cm^2 V^{-1} s^{-1}]**	**Mob. Rev [cm^2 V^{-1} s^{-1}]**	**Mob. Av [cm^2 V^{-1} s^{-1}]**	**V_{th} [V]**	**I_{on}/I_{off}**
**PDPP-TT**	0.57 ± 0.12	0.74 ± 0.14	0.66 ± 0.12	22.69 ± 8	≈ 10^5
**PDPP-TT-PDMS-1000**	0.18 ± 0.009	0.22 ± 0.01	0.21 ± 0.01	29.06 ± 4	≈ 10^6
**PDPP-TT-PDMS-2500**	0.13 ± 0.035	0.17 ± 0.04	0.15 ± 0.04	25.76 ± 2	≈ 10^6
**PDPP-TT-PDMS-25000**	0.07 ± 0.02	0.11 ± 0.03	0.08 ± 0.02	17.08 ± 1	≈ 10^6

4. Conductivity under strain

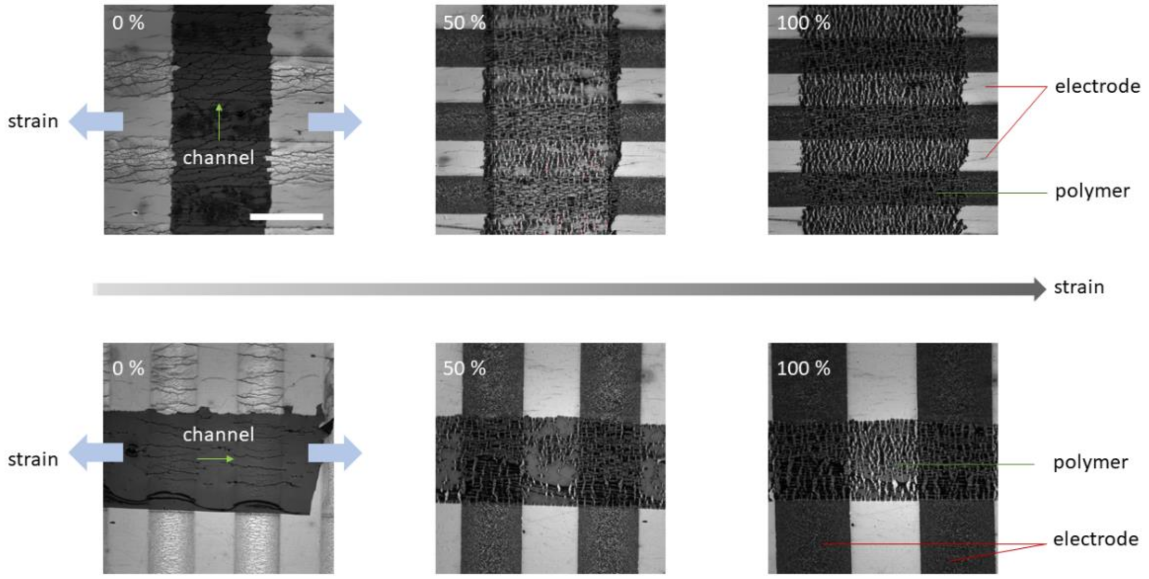

Figure S27. Confocal microscopy image of a PDPP-TT film during the strain-dependent conductivity measurements. Crack formation is observed, and causes a decrease in conductivity. Due to the directionality of the formed cracks with regards to the channel direction (green arrow), the impact on conductivity is different for perpendicular (top) and parallel (bottom) strain directions. Scale bar denotes 1 mm.

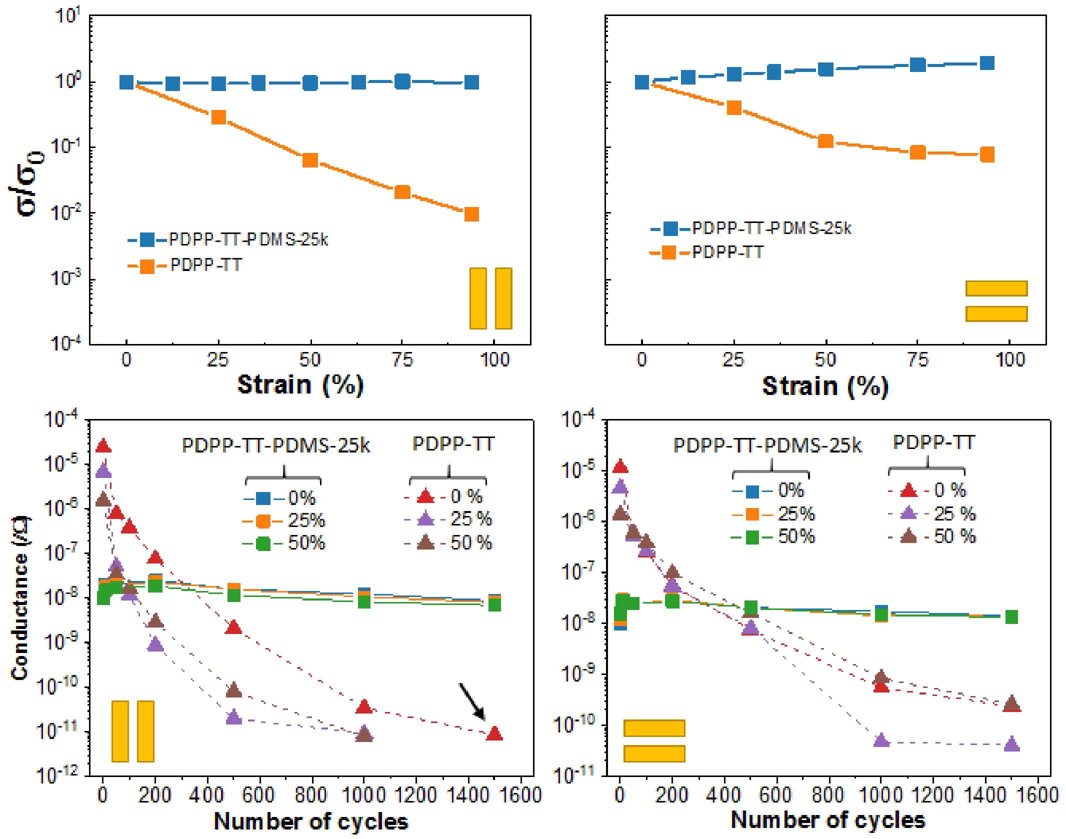

Figure S28. Bulk doping: *top* relative change in DC conductivity of PDPP-TT and PDPP-PDMS-25k under various strains (0% - 100%) with the stretching direction (left) parallel and (right) perpendicular to the channel. *Bottom* DC conductance of PDPP-TT and PDPP-PDMS-25k under various strains (0, 25, 50%) while increasing the number of stretching cycles to 1500. The channel direction is (right) parallel and (left) perpendicular to the stretching direction. Black arrow indicates the disconnection between 2 electrodes.

We also tested a different device setup, and spin-coated the dopant as a layer directly on the films of the semiconducting polymer. Here, disconnection was observed for the reference polymer after parallel stretching at 25% strain. When continuing to strain to 100%, the PDPP-TT film disconnected after less than 50 cycles regardless of the stretching direction and measured strain range (0, 50 and 100%).

The TBC on the other hand maintained relatively stable conductivity values up to 1000 cycles 100% strain, which then decreases almost 1 order of magnitude when continuing to cycle to 100% strain up to 2000 cycles (Figure S29).

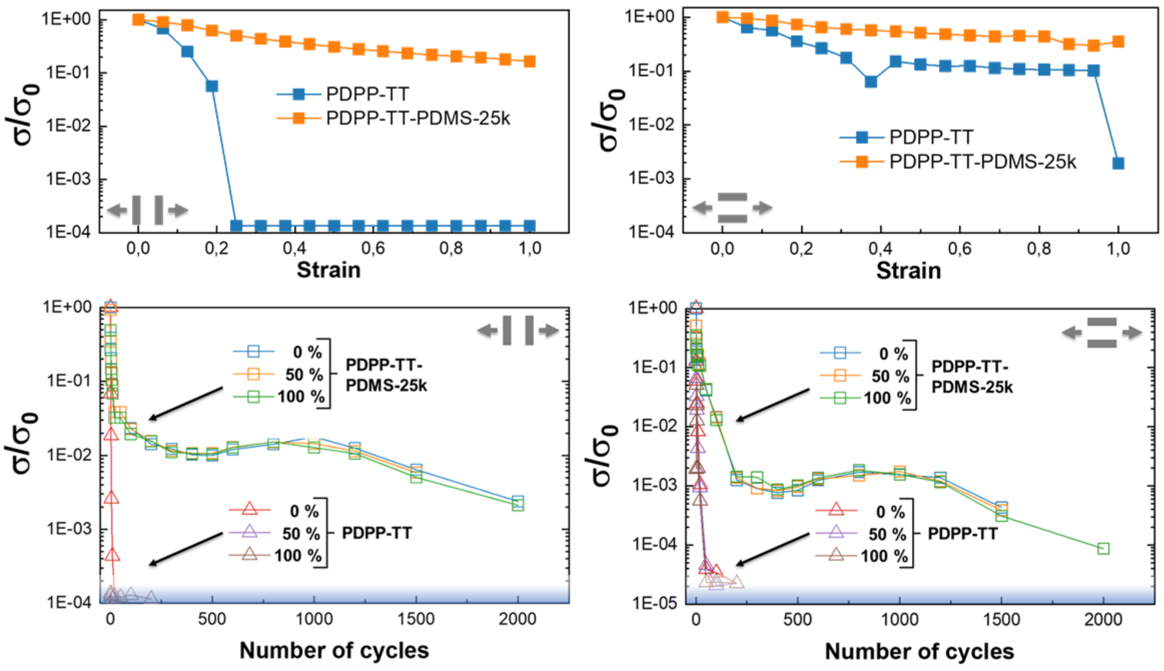

Figure S29. Interfacial doping: *top* relative change in DC conductivity of PDPP-TT and PDPP-PDMS-25k under various strains (0% - 100%) with the stretching direction (left) parallel and (right) perpendicular to the channel. *bottom* relative change in DC conductivity of PDPP-TT and PDPP-PDMS-25k under various strains (0, 50, 100%) while increasing the number of stretching cycles (from ε = 1.0 to ε = 0). The channel direction is (right) parallel and (left) perpendicular to the stretching direction.

5. Low Solvent Processability ("Spreading")

Rheology

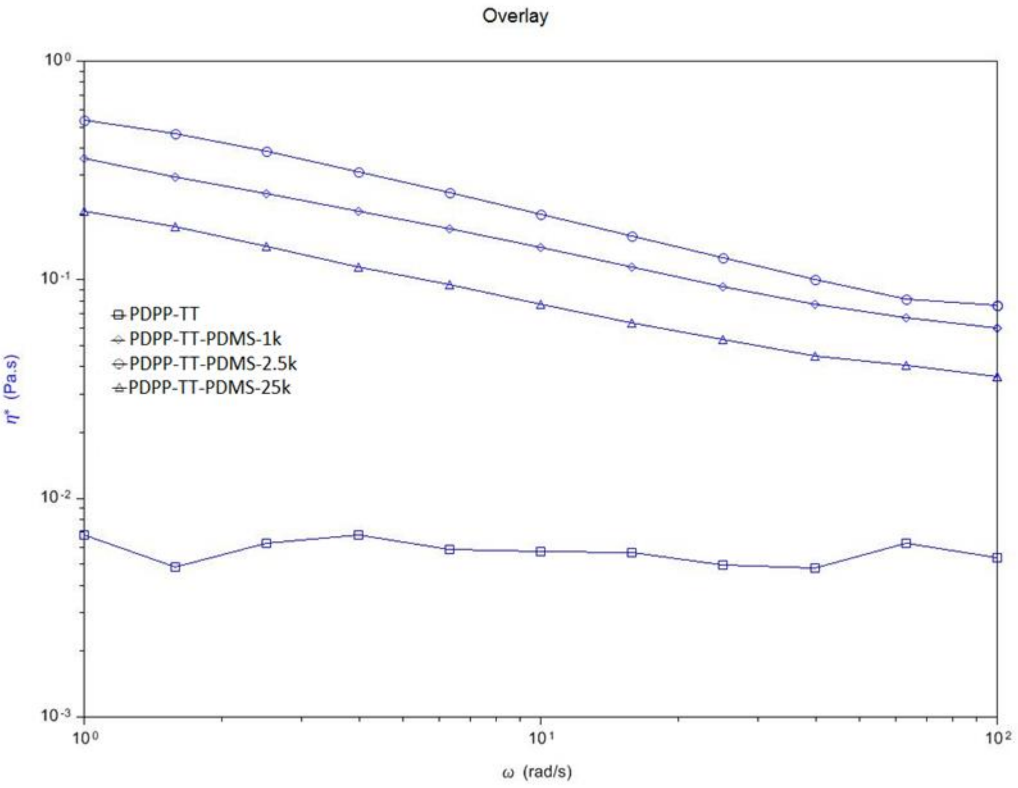

Figure S30. Flow curves of the reference PDPP-TT and block copolymers.

Spreading Experiments

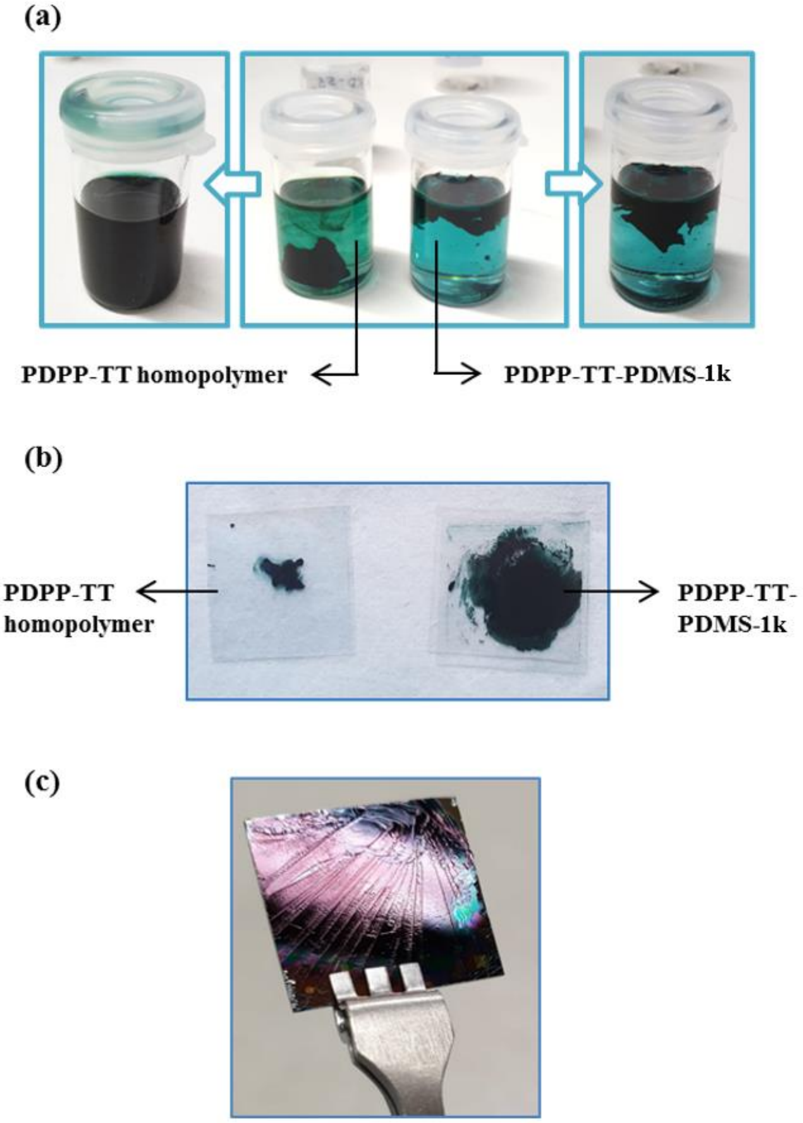

Figure S31. (a) Image demonstrating the swelling ability of PDPP-TT-PDMS-1000 in comparison to the PDPP-TT homopolymer; (b) Image demonstrating different processabilities of PDPP-TT-PDMS-25000 and neat PDPP-TT polymers after adding a drop of chlorobenzene at 120°C; (c) BGBC OFET with spread PDPP-TT-PDMS-1000.

Electrical Characterization: BGBC transfer curves of spread and spin-coated films

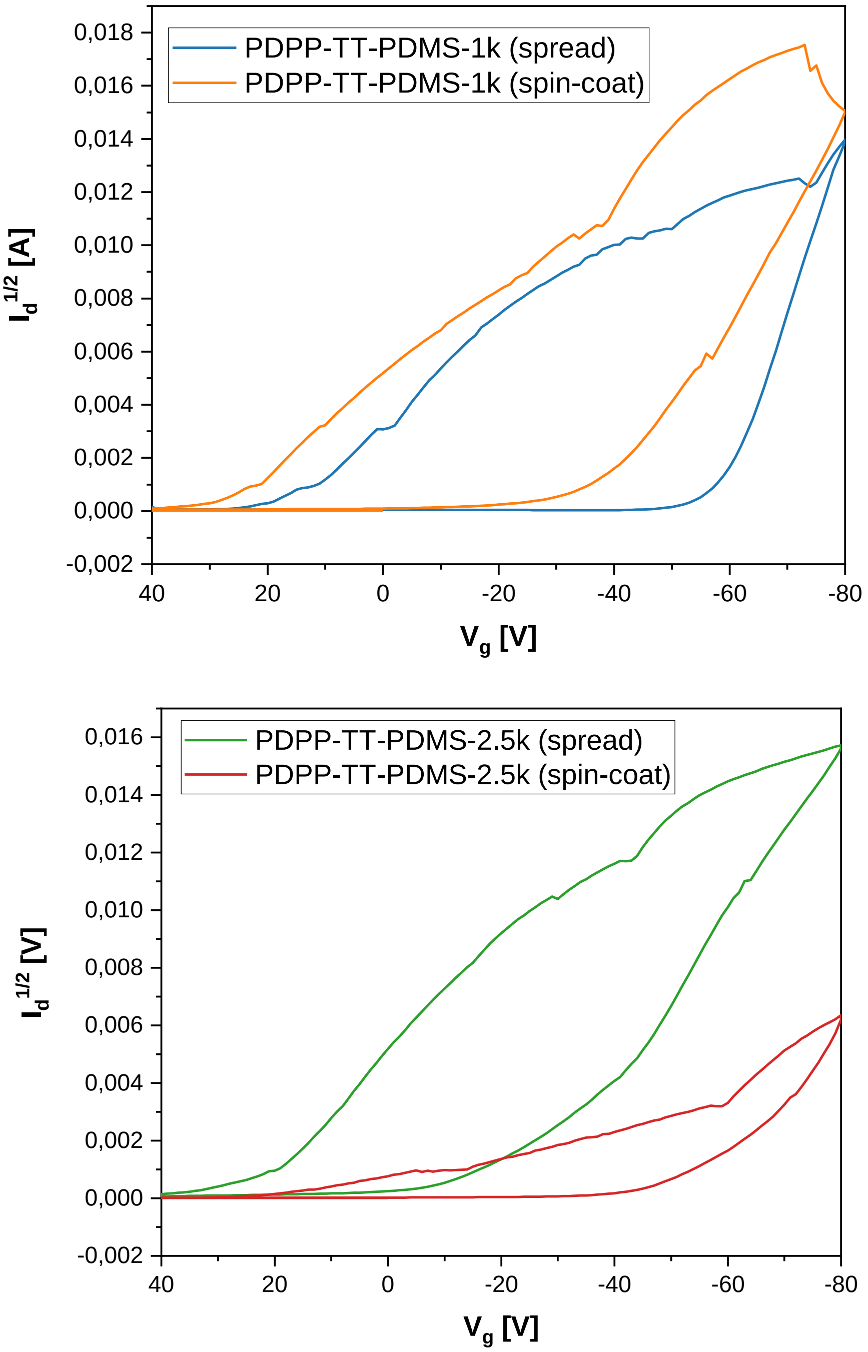

Figure S32. Comparison of the transfer curves of BGBC OFET devices fabricated by spreading and spin-coating: PDPP-TT-PDMS-1k (top) and PDPP-TT-PDMS-2.5k (bottom).

6. References

[1] Y. Ito, A.A. Virkar, S. Mannsfeld, J. H. Oh , M. Toney, J. Locklin, Z. Bao, *J. Am. Chem. Soc.* **2009**, *131*, 9396.

[2] H. H. Choi, K. Cho, C. D. Frisbie, H. Sirringhaus, V. Podzorov, *Nat. Mater.* **2017**, *17*, 2.

[3] J. Yu, B. Zhao, X. Nie, B. Zhou, Y. Li, J. Hai, E. Zhu, L. Bian, H. Wu, W. Tang, *New J. Chem.* **2015**, *39*, 2248.

[4] R. Di Pietro, T. Erdmann, J. H. Carpenter, N. Wang, R. R. Shivhare, P. Formanek, C. Heintze, B. Voit, D. Neher, H. Ade, A. Kiriy, *Chem. Mater.* **2017**, *29*, 10220.
